# A mobile EEG study on the psychophysiological effects of walking and crowding in indoor and outdoor urban environments

**DOI:** 10.1038/s41598-022-20649-y

**Published:** 2022-11-02

**Authors:** Panagiotis Mavros, Michel J Wälti, Mohsen Nazemi, Crystal Huiyi Ong, Christoph Hölscher

**Affiliations:** 1 Singapore-ETH Centre, Future Cities Laboratory, CREATE campus, 1 CREATE Way, #06-01 CREATE Tower, Singapore, 138602 Singapore; 2 Chair of Cognitive Science, Department of Humanities, Social and Political Sciences, ETH Zürich, Zürich, 8092 Switzerland; 3 National University of Singapore, Singapore, Singapore

**Keywords:** Psychology and behaviour, Neuroscience, Environmental social sciences

## Abstract

Environmental psychologists have established multiple psychological benefits of interaction with natural, compared to urban, environments on emotion, cognition, and attention. Yet, given the increasing urbanisation worldwide, it is equally important to understand how differences within different urban environments influence human psychological experience. We developed a laboratory experiment to examine the psychophysiological effects of the physical (outdoor or indoor) and social (crowded versus uncrowded) environment in healthy young adults, and to validate the use of mobile electroencephalography (EEG) and electrodermal activity (EDA) measurements during active walking. Participants (N = 42) were randomly assigned into a walking or a standing group, and watched six 1-min walk-through videos of green, urban indoor and urban outdoor environments, depicting high or low levels of social density. Self-reported emotional states show that green spaces is perceived as more calm and positive, and reduce attentional demands. Further, the outdoor urban space is perceived more positively than the indoor environment. These findings are consistent with earlier studies on the psychological benefits of nature and confirm the effectiveness of our paradigm and stimuli. In addition, we hypothesised that even short-term exposure to crowded scenes would have negative psychological effects. We found that crowded scenes evoked higher self-reported arousal, more negative self-reported valence, and recruited more cognitive and attentional resources. However, in walking participants, they evoked higher frontal alpha asymmetry, suggesting more positive affective responses. Furthermore, we found that using recent signal-processing methods, the EEG data produced a comparable signal-to-noise ratio between walking and standing, and that despite differences between walking and standing, skin-conductance also captured effectively psychophysiological responses to stimuli. These results suggest that emotional responses to visually presented stimuli can be measured effectively using mobile EEG and EDA in ambulatory settings, and that there is complex interaction between active walking, the social density of urban spaces, and direct and indirect affective responses to such environments.

## Introduction

In the next three decades, it is expected that more than 60% of the world’s population will be living in cities^1^. Increasing urban density, rather than sprawl, is promoted by policy-makers and planners due its multiple benefits in terms on sustainability and productivity^2,3^. Meanwhile it is also increasingly clear that urban environments have a major impact on individual and collective psychology and mental health^4^ and that subjective perceptions of environmental quality are strongly associated with well-being^5^. The growing population density can also lead to increased levels of crowding in various types of public urban spaces, especially in public transport facilities^6^ or downtown pedestrian areas^7^. In this context, it is important to understand how the presence of people and pedestrian crowds influence the psychological experience of public spaces^8,9^.

Extensive research during the last decades has demonstrated that compared to urban environments, exposure to nature-based environments—from urban parks to forests—brings multiple psychological benefits on mood, attention and cognitive function^10–12^. However, given the increased urban populations, there is increased interest to move “beyond the nature vs urban dichotomy”^13^ and to study how different types of urban environments influence the psychological state of individuals^14–17^. Multiple physical properties of urban and architectural forms influence psychological perceptions, such as enclosure and spaciousness^18,19^, architectural variation^15^, or even being underground^20^. However, given that urban spaces are often experienced in various levels of social density, or ‘crowding’ it is important to understand how the factor of crowding influences people’s perception of urban spaces. In parallel, although virtual exposure to environments can effectively elicit psychological responses, exposure to the actual environments produces stronger effects^12,21^. While the majority of studies investigating neurophysiological responses to the urban and green environments are conducted in the lab, there are more and more studies conducted outside the laboratory in walking conditions^22–25^, but it is not clear whether walking, as a form of physical activity in itself, influences psychological and neurophysiological responses.

In the field of environmental psychology, a prominent line of research focuses on the psychological restoration of natural versus urban environments^26,27^. According to the Attention–Restoration Theory (ART)^26^, it is postulated that urban environments are often accompanied by high levels of stimulation, e.g. moving cars and people, that require directed attention and lead to directed attention fatigue^28^. In contrast, numerous studies have shown that being in a natural environment, like a park or forest, recruits our attention differently, can alleviate directed attention fatigue, reduce stress^29^, and improve cognitive function^30^. This is also consistent with the Stress Reduction Theory (SRT)^27^, which posits that being exposed to natural (compared to urban) environments reduces physiological and psychological stress which leads to enhanced positive affect. Even exposure to simulated environments of nature, for example in laboratory settings or in virtual reality, is found to be effective in improving mood states^31–33^. Despite numerous studies showing positive psychological effects of natural compared to urban environments, there is some evidence that these effects can be mediated by the aesthetic qualities of the environments presented to participants. Natural environments are not always restorative^31^ and physical attributes linked with the notion of *contemplativeness* (long lines of sight, vegetation quality, landscape design) moderate preference and restoration^31,34^. Moreover, the amount of green features within urban environments^35^, their aesthetic and design quality^36^ and absence of vehicular traffic (i.e. pedestrianised public spaces) can also promote restorative effects that are comparable with those of natural environments^13,16^.

The physical characteristics of the built environment play a major influence on mental health and well-being^37^, however it is not clear if being (or walking) in indoor or outdoor spaces produce similar psychological effects? In the context of pedestrian activity, while a wealth of studies investigated the effects of walking in natural compared to outdoor urban environments, there are fewer studies directly comparing walking in indoor compared to outdoor environments. This question is particularly relevant for cities with extensive indoor and/or underground pedestrian networks, such as Montreal, Hong Kong and Singapore^38^. Previous research tends to study either indoor or outdoor environments separately. The architectural properties of different indoor environments influence how spaces are perceived and how individuals feel^39^. In a variety of settings from domestic interiors to airports, subjective experience and aesthetic judgements are influenced by physical properties such as room width or enclosure type^40^, ceiling height^41^, or ceiling shape, colour and materials^42^. Indoor spaces with windows to dominated nature exterior spaces can improve patient outcomes^43^, and has small effects in improved emotions, thermal comfort, concentration and working memory^44^. Conversely, in the context of outdoor spaces, a wide range of urban design properties have been found to influence subjective experience, including the width of walkways, height of buildings, active land-use adjacent to the walkway^45,46^, or even standing next to high-rise buildings^47^. More than just subjective experience, these properties also influence pedestrian behaviour, such as the propensity of walking, or *walkability*^46^. However, there is a general lack of studies that directly compare whether behaviour or emotions differ between indoor and outdoor environments. For instance, there is evidence that exercising indoors does not produce the same psychological effects as exercising outdoors^48^, and that people may prefer outdoor compared to indoor workplaces^49^. In this context, our study aimed to understand the emotional experience of walking in indoor vs outdoor pedestrian networks, and comparing both with walking in nature as a baseline.

Besides the physical characteristics of space, the social context can also influence people’s subjective experience within a certain environment—for instance, psychological restoration derived from walking in an urban environment increases when accompanied by a friend^50^. Being in the presence of other people is typical for urban and other public spaces, but we can distinguish between the physical property of social density and the psychological experience of *crowding*^51^. Motivated by over-crowding in residential environments^51,52^, earlier studies during the 1970s showed that higher social density is associated with negative emotions^53^, reduces performance in cognitive tasks^52,54^, elevates psychological and physiological stress (e.g. elevated blood pressure levels)^52^, and requires more attentional resources to focus on pertinent information^55^. Different theories were proposed to explain these findings, such as excessive social stimulation^53^, or lack of control^55^. However, these early studies involved static situations, such as sitting in a room and completing various cognitive tests^55–57^. However, despite the overall negative associations of crowding^6,58^, there are cases where high social density is perceived as desirable, such as increased satisfaction derived from being in a busy retail environment^59,60^ or willingness to walk on a street^61^. The social characteristics of the crowd influence how individuals feel, walk (for a review of recent crowd research^62^) or navigate^17,63^. Potentially active movement may preserve feelings of control. In the presence of a pedestrian crowd, people spontaneously adjust their proximity and speed in order to walk in closer proximity to perceived group members and to experience less discomfort^64–66^. The physical and social characteristics of space can also interact, and jointly influence overall subjective experience (e.g. perceptions of comfort and agency)^67^ and collective behaviour. For instance, in a crowded space, comfort increases when individuals have visual access to outdoors^6^, or having a view of the sky^68^.

The psychological and cognitive effects of exposure to different types of environments can be assessed with a variety of different measures. In the present study, we combine self-reported measures with electroencephalography (EEG) and electrodermal activity (EDA), two types of physiological measures of stress, attention, emotion and cognition that are well established in environmental psychophysiology^69^. EEG, a measure of the electrical potentials produced by the coordinated firing of neurons in the cortex, is used to measure changes in brain activity in response to stimuli of natural and urban environments^24,29,34,43,70^. EDA (or skin conductance; SC) is controlled by the autonomic nervous system and is used as indicator of physiological arousal^71,72^. EDA has been widely used in emotion and attention restoration studies^73,74^ to measure and visualise subjective experience of pedestrians in virtual reality^75^ or in the real world^23,76,77^, the experience of mobility of older people^78^, or stress while cycling in urban environments^79,80^.

While many of the above behavioural studies on the psychological effects of environments (e.g. attention restoration) have been conducted both in laboratory environments and real-world settings, the majority of psychophysiological studies comparing urban versus green environments, have been conducted in laboratory conditions^43,81^ which enable close experimental control. In such settings, exposure to different environments is achieved through the presentation of image slideshows, videos and more recently immersive 360-degree videos in virtual reality^82^. The effort to study “cognition in action”^83^ during the last decades has greatly advanced from the development of mobile electrophysiological recording devices, frameworks for mobile brain imaging such as MoBI^84^, and new signal processing tools^85–87^. Mobile EEG acquisition and signal processing techniques have been validated, e.g. detecting event-related potentials (ERP) during an auditory oddball task^88,89^ or observing steady-state visually evoked potentials (SSVEP)^90^. As a result, mobile EEG studies are now feasible and participants can experience different environments while walking either inside the laboratory^91,92^ or ‘in-the-wild’^87,89,93–96^. In the context of environmental psychology, studies have also taken place outside the laboratory while participants walk^22,24^ or sit^25^. However, walking, as a form of physical activity has multiple effects on cognition and attention, which may influence the psychological impact of different environments compared to being exposed to the same environment while being immobile. Although spatial working memory is not influenced by walking^93^, walking enhances the processing of peripheral visual information^90^, which could partially be attributed to increased demands of attentional processing of moving through space (i.e. visual flow) and not walking per se^97^. These studies also find that these attentional demands of walking through space are associated with attenuated EEG power, e.g. lower alpha power^90^. Furthermore, as a form of physical activity, walking itself can have positive psychological effects^98^. Finally, there is a potential moderating role of the environment that should be taken into account, because the salutogenic effects of walking (stress reduction, attention restoration, and cognitive function) may be stronger in green and blue environments than in urban ones^32,99^.

To summarise, although it has been demonstrated that exposure to green—compared to urban—spaces is psychologically beneficial, it is unclear how different types of urban spaces, in particularly indoor versus outdoor influence individuals, and further, how the levels of occupancy influence how spaces are experienced. In the present study, we modified a naturalistic laboratory-based environmental exposure paradigm from environmental psychology, and we asked participants to watch six videos of walking through urban walkways, while they walked or stood on a treadmill. The videos included urban indoor and urban outdoor spaces in low and high levels of crowding, as well as a green space. We measured their subjective experience using self-reports (self-assessment manikin)^100^, their brain activity using mobile electroencephalography (EEG) and their physiological arousal using mobile electrodermal activity (EDA). This experimental design allows us to assess the psychological effects of the environmental stimuli as well as the effects of active walking on emotions and on physiological signals.

Earlier studies have demonstrated a positive relationship between attention restoration, cognitive function, and positive affect after exposure to natural, as opposed to urban environments. We therefore hypothesize that overall, exposure to the green scenes will result in more positive valence, lower arousal, and lower attentional demand (self-reports, EEG, EDA) than indoor or outdoor urban spaces. Given fundamental differences in their spatial characteristics, for example in terms of spaciousness, boundary height, or sense of enclosure, we also hypothesize that videos of outdoor urban spaces will result in more positive valence, and lower arousal (self-reports, EEG, EDA) compared to indoor spaces. We further hypothesize that videos of crowded spaces will result in more negative valence and higher arousal (self-reports, EEG, EDA) compared to uncrowded indoor or outdoor urban spaces. In an alternative hypothesis, if pedestrian crowds are associated with human occupancy and urban vibrancy, crowded scenes will evoke more positive responses. Finally, to the best of our knowledge, a systematic comparison of mobile EEG during standing or walking has not been tested so far in the context of measuring environmental exposure. Hence, it is unknown how much physical activity impacts related neurophysiological measures or subjective experience. We hypothesize that walking will result in increased noise in the signal as well as intensify self-reported emotions.

## Results

### Manipulation check

Table 1 shows the results of *bayesian directional hypothesis tests* to evaluate whether walking, compared to standing, influenced physiological measurements (EEG or EDA), or the psychological experience of spatial presence. We found did not find evidence for a difference between the standing and the walking groups in terms of perceived spatial presence (MEC-SPQ, $posterior~ probability = 0.49$). However, we found a trend for a difference between the two groups in terms of the EEG signal-to-noise ratio (SNR; $posterior~ probability = 0.88$). Note that SNR values were calculated on cleaned datasets (i.e., after removing rejected artefact IC’s). In terms of the EDA measures, we found strong evidence that skin conductance levels (SCL; $posterior~ probability = 1.0$) and non-specific skin conductance responses (nSCR: $posterior~ probability = 1.0$) were higher in the walking group, and substantial evidence for a difference on integrated skin conductance responses (ISCR: $posterior~ probability = 0.94$).

**Table 1:** Hypothesis tests on the effects of walking.

Measure	Variable	Hypothesis	Estimate	Est. error	95% CI	Evid.Ratio	Post.Prob	Star
EEG	SNR	(GroupWalking)> 0	1.004	0.865	[− 0.445, 2.438]	7.15	0.88	
EDA	SCL	(GroupWalking)> 0	0.337	0.101	[0.169, 0.503]	3999.00	1.00	*
	ISCR	(GroupWalking)> 0	0.513	0.331	[− 0.030, 1.045]	15.39	0.94	
Presence	MEC_SPQ	(GroupWalking) > 0	0.001	0.195	[− 0.318, 0.334]	0.97	0.49	

*EEG* electroencephalography, *SNR* signal-to-noise ratio, *EDA* electrodermal activity, *SCL* skin conductance levels, *ISCR* integrated skin conductance response.

### Effects of environments

**Figure 1:**
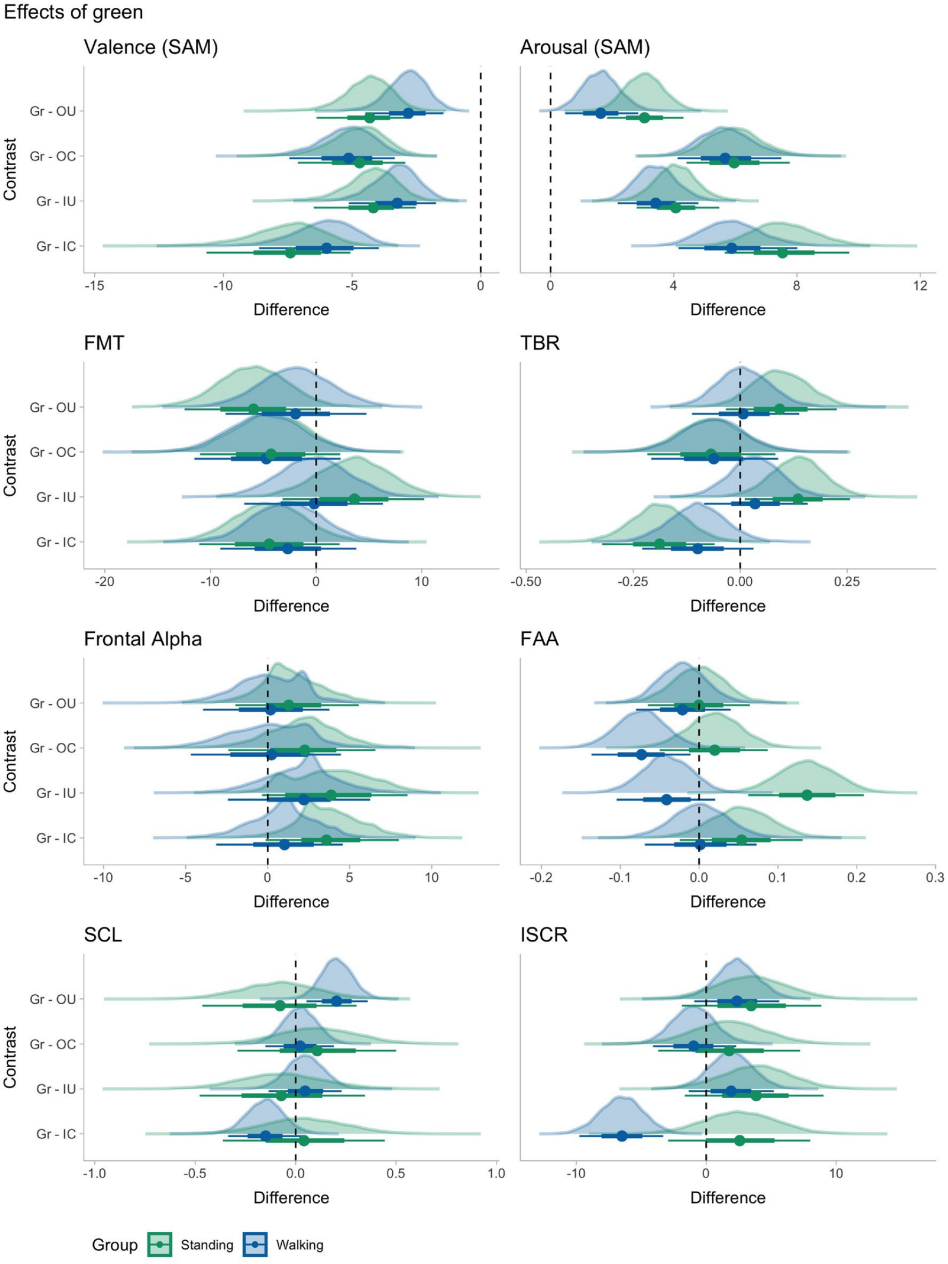
Posterior density plots of pairwise contrasts between the green and the urban scenes (outdoor and indoor). For each parameter estimate the dot represents the median, and horizontal bars represent then 66% (thick) and 95% (thin) credible intervals.

**Figure 2:**
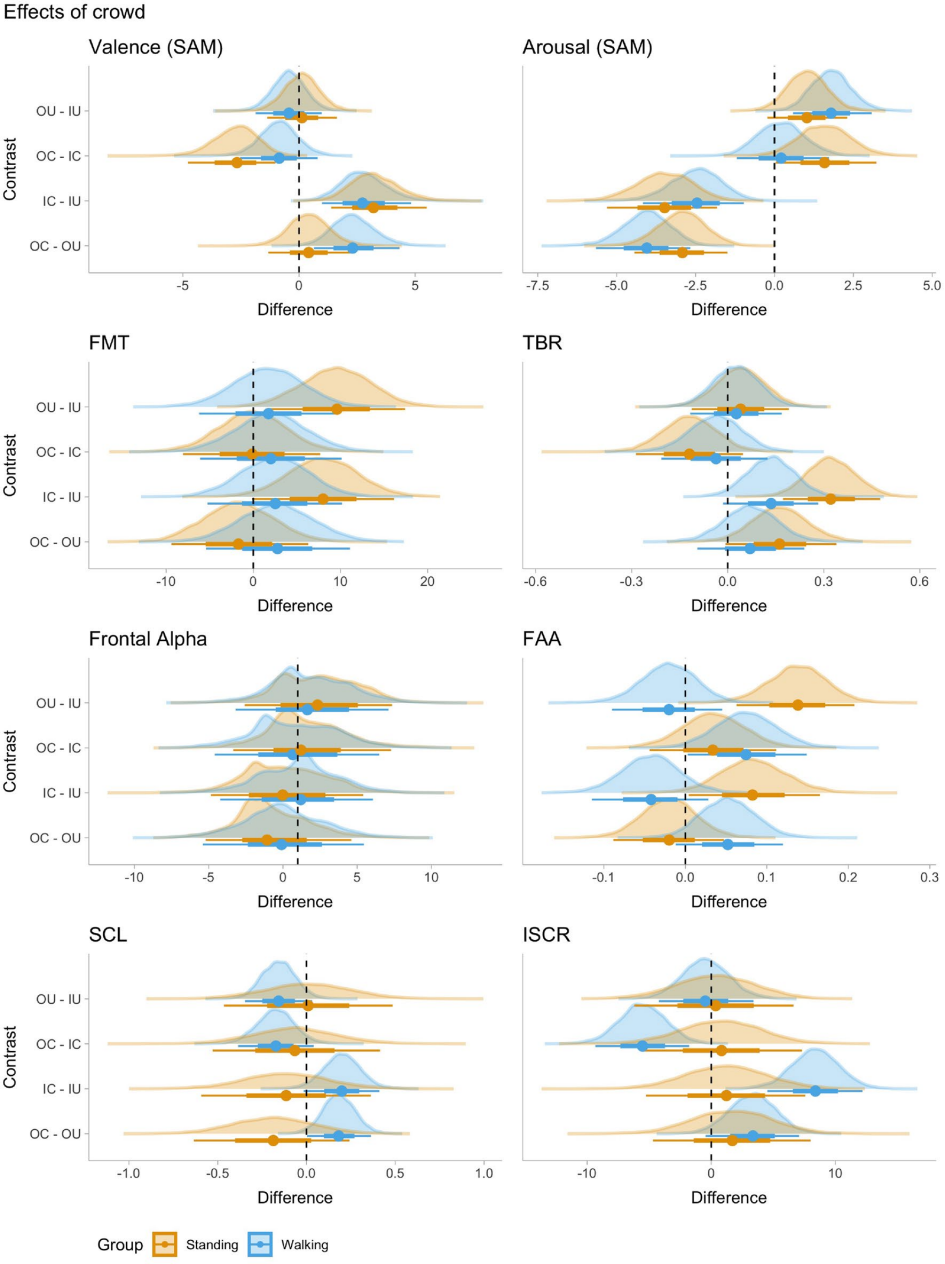
Posterior density plots of pairwise contrasts between the crowded and uncrowded scenes. For each parameter estimate the dot represents the median, and horizontal bars represent then 66% (thick) and 95% (thin) credible intervals.

To evaluate the effects of the presented video stimuli, we fitted separate Bayesian hierarchical regression models for each measure. Subsequently we performed pairwise contrast analyses by estimating marginal means between the types of environments (green, indoor, outdoor) and social density (crowded, uncrowded). Figure 1 presents the results of the planned contrasts between green and urban scenes, while Fig. 2 shows planned contrasts between crowded and non-crowded scenes. In the following subsections, we present the results of the planned contrast analysis for each variable, while the model estimates are included in the Supplementary Materials.

### Self-reported arousal and valence

Tables 2 and 3 show the contrasts performed; the model estimates can be found in the Supplementary Materials. For clarity, given that higher SAM ratings correspond to more calm or more negative self-reported emotion, a positive contrast suggests that the first parameter had higher SAM ratings; e.g. for *arousal* if the contrast is $Gr-OC> 0$ would suggest that the green space was perceived as more calm, while for *valence* if the contrast is $Gr-OC < 0$ would suggest that the ‘outdoor crowded‘ environment had a more negative (higher) rating than the green.

**Table 2:** Contrasts for self-reported arousal, measured using the SAM.

Contrast	Group	Median	SD	95% CI	PP	ER01	ER10	Star
**Gr–IC**	**Stand**	**7.529**	**1.036**	**[5.577,9.579]**	**1.000**	**Inf**	**0.000**	*
**Gr–IC**	**Walk**	**5.879**	**0.984**	**[4.117,7.942]**	**1.000**	**Inf**	**0.000**	*
**Gr–IU**	**Stand**	**4.064**	**0.673**	**[2.792,5.475]**	**1.000**	**Inf**	**0.000**	*
**Gr–IU**	**Walk**	**3.412**	**0.666**	**[2.114,4.711]**	**1.000**	**Inf**	**0.000**	*
**Gr–OC**	**Stand**	**5.958**	**0.855**	**[4.279,7.617]**	**1.000**	**Inf**	**0.000**	*
**Gr–OC**	**Walk**	**5.665**	**0.860**	**[4.083,7.433]**	**1.000**	**Inf**	**0.000**	*
**Gr–OU**	**Stand**	**3.047**	**0.636**	**[1.806,4.279]**	**1.000**	**Inf**	**0.000**	*
**Gr–OU**	**Walk**	**1.627**	**0.606**	**[0.421,2.786]**	**0.998**	**420.053**	**0.002**	*
**OC–OU**	**Stand**	**− 2.919**	**0.752**	**[− 4.432, − 1.484]**	**0.000**	**0.000**	**Inf**	*
**OC–OU**	**Walk**	**− 4.046**	**0.755**	**[− 5.501, − 2.563]**	**0.000**	**0.000**	**Inf**	*
**IC–IU**	**Stand**	**− 3.482**	**0.896**	**[− 5.202, − 1.72]**	**0.000**	**0.000**	**Inf**	*
**IC–IU**	**Walk**	**− 2.454**	**0.814**	**[− 4.136, − 0.946]**	**0.001**	**0.001**	**887.889**	*
**OC–IC**	**Stand**	**1.586**	**0.817**	**[0.036,3.232]**	**0.976**	**41.328**	**0.024**	*
OC–IC	Walk	0.210	0.753	[− 1.188, 1.729]	0.606	1.539	0.650	
**OU–IU**	**Stand**	**1.028**	**0.640**	**[− 0.255,2.258]**	**0.948**	**18.185**	**0.055**	
**OU–IU**	**Walk**	**1.794**	**0.634**	**[0.598,3.09]**	**0.998**	**532.333**	**0.002**	*

Note that arousal was measured in a 9-point scale (1 = excited to 9 = calm).

Median, standard deviation (SD), 95% credible intervals (CI) of contrasts between posterior estimates, Posterior probability (PP), evidence ratio (ER), ER01 in favour of the directional hypothesis (PP> 0) and ER10 in favour of the altenrative hypothesis (PP < 0), bold = posteior probability exceeds 85%; star = posterior probability exceeds 95%.

For self-reported arousal, contrast analysis revealed evidence that green space was perceived as more calming (i.e. higher ratings) than either indoor or outdoor spaces. The effect was higher in contrasts between green and crowded scenes. Comparing the urban environments, the outdoor spaces were perceived as more calm than indoor spaces for each level of crowding (OC> IC; OU> IU), apart from the walking condition and uncrowded scenes. Finally, both indoor and outdoor crowded spaces were less calming than uncrowded spaces (OC < OU; IC < IU).

**Table 3:** Contrasts for self-reported valence (SAM).

Contrast	Group	Median	SD	95% CI	PP	ER01	ER10	Star
**Gr–IC**	**Stand**	**− 7.397**	**1.419**	**[− 10.441, − 4.916]**	**0.000**	**0.000**	**Inf**	*
**Gr–IC**	**Walk**	**− 5.987**	**1.200**	**[− 8.508, − 3.895]**	**0.000**	**0.000**	**Inf**	*
**Gr–IU**	**Stand**	**− 4.174**	**0.985**	**[− 6.267, − 2.388]**	**0.000**	**0.000**	**Inf**	*
**Gr–IU**	**Walk**	**− 3.236**	**0.862**	**[− 5.042, − 1.675]**	**0.000**	**0.000**	**Inf**	*
**Gr–OC**	**Stand**	**− 4.706**	**1.068**	**[− 6.973, − 2.83]**	**0.000**	**0.000**	**Inf**	*
**Gr–OC**	**Walk**	**− 5.132**	**1.045**	**[− 7.403, − 3.332]**	**0.000**	**0.000**	**Inf**	*
**Gr–OU**	**Stand**	**− 4.311**	**0.917**	**[− 6.246, − 2.673]**	**0.000**	**0.000**	**Inf**	*
**Gr–OU**	**Walk**	**− 2.809**	**0.766**	**[− 4.387, − 1.405]**	**0.000**	**0.000**	**Inf**	*
OC–OU	Stand	0.415	0.897	[− 1.393, 2.156]	0.681	2.131	0.469	
**OC–OU**	**Walk**	**2.311**	**0.926**	**[0.546,4.195]**	**0.996**	**274.862**	**0.004**	*
**IC–IU**	**Stand**	**3.203**	**1.050**	**[1.36,5.465]**	**1.000**	**7999.000**	**0.000**	*
**IC–IU**	**Walk**	**2.733**	**0.980**	**[0.96,4.784]**	**0.999**	**999.000**	**0.001**	*
**OC–IC**	**Stand**	**− 2.670**	**0.956**	**[− 4.7, − 0.963]**	**0.001**	**0.001**	**1141.857**	*
**OC–IC**	**Walk**	**− 0.853**	**0.834**	**[− 2.504, 0.83]**	**0.142**	**0.165**	**6.067**	
OU–IU	Stand	0.123	0.758	[− 1.424, 1.554]	0.570	1.324	0.756	
OU–IU	Walk	− 0.434	0.724	[− 1.846, 0.999]	0.269	0.368	2.721	

Note that valence was measured on a 9-point scale (1 = positive and 9 = negative).

Median, standard deviation (SD), 95% credible intervals (CI) of contrasts between posterior estimates, Posterior probability (PP), evidence ratio (ER), ER01 in favour of the directional hypothesis (PP> 0) and ER10 in favour of the altenrative hypothesis (PP < 0), bold = posteior probability exceeds 85%; star = posterior probability exceeds 95%.

For self-reported valence, we found that green space is perceived more positively (i.e., lower rating on the 1–9 scale) than either the indoor spaces or the outdoor spaces (IC/IU/OC/OU < Gr). Crowded spaces (either indoor or outdoor) were perceived more negatively than the uncrowded spaces (IC> IU; OC> OU), apart from the outdoor space for the walking group. Last, the indoor urban spaces were perceived more negatively compared to outdoor urban spaces (OC < IC) in the crowded condition, but there was no difference in valence in the uncrowded case (i.e. OU/IU).

### Physiological results

#### Frontal alpha power

For reference with earlier studies in the restorativeness of nature-based environments, we report alpha power averaged over the frontal electrodes (see “Methods”). Table 4 shows contrasts for frontal alpha power. We found that the green environment led to higher alpha power than urban indoor and outdoor (both crowded and uncrowded), but the evidence for an effect was substantial only for the standing group. Contrasts between the urban environments revealed a trend for higher alpha in non-crowded environments, and in outdoor compared to indoor, but the evidence was anecdotal.

**Table 4:** Contrasts for frontal alpha (FA).

Contrast	Group	Median	SD	95% CI	PP	ER01	ER10	Star
Gr–IC	Walk	1.024	1.939	[− 3.004, 4.696]	0.717	2.534	0.395	
**Gr–IC**	**Stand**	**3.583**	**2.002**	**[− 0.366, 7.707]**	**0.970**	**32.473**	**0.031**	*
Gr–IU	Walk	2.201	2.154	[− 2.426, 6.241]	0.826	4.743	0.211	
**Gr–IU**	**Stand**	**3.869**	**2.451**	**[− 0.298, 8.539]**	**0.966**	**28.304**	**0.035**	*
Gr–OC	Walk	0.231	2.385	[− 4.693, 4.455]	0.542	1.182	0.846	
Gr–OC	Stand	2.260	2.227	[− 2.354, 6.575]	0.846	5.472	0.183	
Gr–OU	Walk	0.173	1.992	[− 3.931, 3.762]	0.533	1.142	0.876	
Gr–OU	Stand	1.281	1.858	[− 2.314, 5.116]	0.811	4.295	0.233	
OC–OU	Walk	− 0.095	2.698	[− 5.457, 5.333]	0.485	0.941	1.062	
OC–OU	Stand	− 1.066	2.427	[− 5.148, 4.635]	0.344	0.526	1.903	
IC–IU	Walk	1.199	2.557	[− 4.084, 6.145]	0.672	2.048	0.488	
IC–IU	Stand	− 0.017	2.732	[− 4.825, 5.426]	0.497	0.989	1.012	
OC–IC	Walk	0.663	2.841	[− 4.405, 6.616]	0.576	1.356	0.738	
OC–IC	Stand	1.207	2.597	[− 3.639, 6.834]	0.723	2.610	0.383	
OU–IU	Walk	1.643	2.603	[− 2.846, 7.437]	0.768	3.303	0.303	
OU–IU	Stand	2.332	2.652	[− 2.507, 7.404]	0.808	4.208	0.238	

Median, standard deviation (SD), 95% credible intervals (CI) of contrasts between posterior estimates, Posterior probability (PP), evidence ratio (ER), ER01 in favour of the directional hypothesis (PP> 0) and ER10 in favour of the altenrative hypothesis (PP < 0), bold = posteior probability exceeds 85%; star = posterior probability exceeds 95%.

#### Frontal midline theta (FMT)

Table 5 shows contrasts for frontal midline theta. We found strong evidence that FMT was higher in the indoor crowded compared to green for the standing group, and higher in the urban outdoor excess for walking in the outdoor uncrowded. We also found that for the standing group, the indoor crowded evoked higher FMT than the indoor uncrowded, and the outdoor uncrowded higher than the indoor uncrowded. Evidence for all other contrasts was anecdotal (evidence ratio < 3).

**Table 5:** Contrasts for frontal midline theta (FMT).

Contrast	Group	Median	SD	95% CI	PP	ER01	ER10	Star
Gr–IC	Walk	− 2.219	2.745	[− 7.344, 3.342]	0.208	0.263	3.808	
**Gr–IC**	**Stand**	**− 4.168**	**3.090**	**[− 10.245, 1.702]**	**0.088**	**0.097**	**10.331**	
Gr–IU	Walk	0.524	2.796	[− 5.103, 5.925]	0.577	1.361	0.735	
Gr–IU	Stand	2.952	3.177	[− 3.015, 9.552]	0.824	4.666	0.214	
**Gr–OC**	**Walk**	**− 3.667**	**2.891**	**[− 9.372, 1.91]**	**0.100**	**0.111**	**8.988**	
**Gr–OC**	**Stand**	**− 4.519**	**3.202**	**[− 10.846, 1.554]**	**0.076**	**0.082**	**12.180**	
Gr–OU	Walk	− 1.978	2.768	[− 7.756, 3.093]	0.236	0.310	3.231	
**Gr–OU**	**Stand**	**− 5.368**	**2.951**	**[− 11.22, 0.297]**	**0.036**	**0.038**	**26.586**	*
OC–OU	Walk	1.743	3.556	[− 5.271, 8.756]	0.688	2.200	0.455	
OC–OU	Stand	− 0.837	3.894	[− 7.995, 7.209]	0.414	0.708	1.413	
IC–IU	Walk	2.801	3.433	[− 3.95, 9.504]	0.793	3.828	0.261	
**IC–IU**	**Stand**	**7.063**	**3.761**	**[− 0.662, 14.147]**	**0.968**	**30.373**	**0.033**	*
OC–IC	Walk	1.511	3.474	[− 5.328, 8.332]	0.662	1.956	0.511	
OC–IC	Stand	0.284	3.842	[− 6.917, 8.252]	0.535	1.149	0.870	
OU–IU	Walk	2.510	3.436	[− 4.357, 9.14]	0.769	3.331	0.300	
**OU–IU**	**Stand**	**8.331**	**3.699**	**[1.085,15.424]**	**0.987**	**75.190**	**0.013**	*

Median, standard deviation (SD), 95% credible intervals (CI) of contrasts between posterior estimates, Posterior probability (PP), evidence ratio (ER), ER01 in favour of the directional hypothesis (PP> 0) and ER10 in favour of the altenrative hypothesis (PP < 0), bold = posteior probability exceeds 85%; star = posterior probability exceeds 95% .

#### Theta/beta ratio (TBR)

Table 6 presents contrasts for theta/beta ratio (TBR). We found decisive evidence for higher TBR in the crowded space (both outdoor and indoor) compared to the uncrowded scenes, except for the walking group and urban outdoor. TBR was higher in the indoor crowded compared to green for both groups, but higher in the green (vs indoor uncrowded and the outdoor uncrowed) for the standing group. TBR was higher in the crowded condition, apart from the outdoor for the walking group. It was also higher for the indoor crowded compared to outdoor crowded for the standing group. Evidence for all other contrasts was anecdotal (evidence ratio < 3).

**Table 6:** Contrasts for theta beta ratio (TBR).

Contrast	Group	Median	SD	95% CI	PP	ER01	ER10	Star
**Gr–IC**	**Walk**	**− 0.099**	**0.066**	**[− 0.233, 0.026]**	**0.067**	**0.072**	**13.953**	
**Gr–IC**	**Stand**	**− 0.188**	**0.067**	**[− 0.322, − 0.06]**	**0.003**	**0.003**	**379.952**	*
Gr–IU	Walk	0.035	0.061	[− 0.079, 0.162]	0.724	2.628	0.381	
**Gr–IU**	**Stand**	**0.135**	**0.062**	**[0.015,0.261]**	**0.982**	**53.795**	**0.019**	*
Gr–OC	Walk	− 0.062	0.075	[− 0.213, 0.082]	0.194	0.240	4.168	
Gr–OC	Stand	− 0.068	0.076	[− 0.212, 0.087]	0.179	0.217	4.598	
Gr–OU	Walk	0.007	0.063	[− 0.115, 0.135]	0.550	1.221	0.819	
**Gr–OU**	**Stand**	**0.093**	**0.067**	**[− 0.035, 0.224]**	**0.929**	**13.011**	**0.077**	
OC–OU	Walk	0.070	0.085	[− 0.093, 0.241]	0.804	4.102	0.244	
**OC–OU**	**Stand**	**0.162**	**0.087**	**[0,0.344]**	**0.972**	**34.242**	**0.029**	*
**IC–IU**	**Walk**	**0.135**	**0.075**	**[− 0.006, 0.289]**	**0.963**	**25.846**	**0.039**	*
**IC–IU**	**Stand**	**0.322**	**0.078**	**[0.172,0.475]**	**1.000**	**Inf**	**0.000**	*
OC–IC	Walk	− 0.037	0.084	[− 0.199, 0.133]	0.328	0.489	2.046	
**OC–IC**	**Stand**	**− 0.120**	**0.085**	**[− 0.285, 0.048]**	**0.074**	**0.080**	**12.491**	
OU–IU	Walk	0.027	0.073	[− 0.114, 0.171]	0.643	1.798	0.556	
OU–IU	Stand	0.040	0.077	[− 0.108, 0.194]	0.703	2.371	0.422	

Median, standard deviation (SD), 95% credible intervals (CI) of contrasts between posterior estimates, Posterior probability (PP), evidence ratio (ER), ER01 in favour of the directional hypothesis (PP> 0) and ER10 in favour of the altenrative hypothesis (PP < 0), bold = posteior probability exceeds 85%; star = posterior probability exceeds 95%.

#### Frontal alpha asymmetry

Table 7 presents contrasts for frontal alpha asymmetry (FAA). Note that higher FAA indicates greater relative left frontal cortical activation, which is associated with more positive/approach affective states (see “Methods”). Overall, we found that for the standing group FAA was higher in the green compared to indoor crowded and indoor uncrowded; but for the walking group FAA was higher in the outdoor crowded and indoor uncrowded compared to the green.

**Table 7:** Contrasts for frontal alpha asymmetry (FAA).

Contrast	Group	Median	SD	95% CI	PP	ER01	ER10	Star
Gr–IC	Walk	0.001	0.036	[− 0.07, 0.071]	0.518	1.073	0.932	
**Gr–IC**	**Stand**	**0.054**	**0.040**	**[− 0.025, 0.131]**	**0.915**	**10.765**	**0.093**	
**Gr–IU**	**Walk**	**− 0.041**	**0.032**	**[− 0.106, 0.019]**	**0.095**	**0.105**	**9.499**	
**Gr–IU**	**Stand**	**0.137**	**0.038**	**[0.063,0.209]**	**1.000**	**3999.000**	**0.000**	*
**Gr–OC**	**Walk**	**− 0.073**	**0.032**	**[− 0.135, − 0.009]**	**0.011**	**0.012**	**86.912**	*
Gr–OC	Stand	0.020	0.035	[− 0.051, 0.085]	0.719	2.563	0.390	
Gr–OU	Walk	− 0.021	0.030	[− 0.081, 0.038]	0.239	0.314	3.186	
Gr–OU	Stand	0.000	0.033	[− 0.066, 0.063]	0.496	0.984	1.016	
**OC–OU**	**Walk**	**0.052**	**0.034**	**[− 0.009, 0.122]**	**0.946**	**17.605**	**0.057**	
OC–OU	Stand	− 0.020	0.034	[− 0.089, 0.047]	0.272	0.375	2.670	
**IC–IU**	**Walk**	**− 0.042**	**0.036**	**[− 0.116, 0.026]**	**0.113**	**0.127**	**7.869**	
**IC–IU**	**Stand**	**0.082**	**0.041**	**[0.008,0.169]**	**0.980**	**48.689**	**0.021**	*
**OC–IC**	**Walk**	**0.074**	**0.038**	**[0.001,0.147]**	**0.980**	**47.780**	**0.021**	*
OC–IC	Stand	0.034	0.039	[− 0.044, 0.111]	0.812	4.326	0.231	
OU–IU	Walk	− 0.020	0.034	[− 0.088, 0.047]	0.278	0.386	2.591	
**OU–IU**	**Stand**	**0.138**	**0.037**	**[0.064,0.209]**	**1.000**	**1999.000**	**0.001**	*

Median, standard deviation (SD), 95% credible intervals (CI) of contrasts between posterior estimates, Posterior probability (PP), evidence ratio (ER), ER01 in favour of the directional hypothesis (PP> 0) and ER10 in favour of the altenrative hypothesis (PP < 0), bold = posteior probability exceeds 85%; star = posterior probability exceeds 95%.

Furthermore, when looking on the effect of crowding we found that FAA was higher in the outdoor crowded compared to uncrowded but higher in the uncrowded compared to crowded for the walking group, whereas for the standing group FAA was higher in the indoor crowded compared to uncrowded. In terms of responses to the urban environments, FAA was higher in the outdoor compared to indoor (both crowded and uncrowded) for the standing group, but for the walking group FAA was higher during the indoor uncrowded compared to outdoor uncrowded.

#### Skin conductance levels (SCL)

Table 8 presents contrasts for skin conductance levels (i.e. Tonic EDA). For the walking group, we found that SCL was higher in the indoor crowded compared to green, but higher in the green compared to outdoor uncrowded. Also for the walking group SCL was higher in the in the crowded compared to the uncrowded conditions (both for indoor and outdoor) and higher in the indoor compared to the outdoor conditions. Contrasts for the standing group were unclear.

**Table 8:** Contrasts for skin conductance levels (SCL).

Contrast	Group	Median	SD	95% CI	PP	ER01	ER10	Star
Gr–IC	Stand	0.042	0.206	[− 0.376, 0.43]	0.583	1.400	0.715	
**Gr–IC**	**Walk**	**− 0.150**	**0.093**	**[− 0.324, 0.042]**	**0.050**	**0.053**	**18.900**	
Gr–IU	Stand	− 0.072	0.211	[− 0.478, 0.345]	0.359	0.559	1.788	
Gr–IU	Walk	0.047	0.093	[− 0.128, 0.234]	0.693	2.260	0.442	
Gr–OC	Stand	0.107	0.199	[− 0.269, 0.515]	0.710	2.444	0.409	
Gr–OC	Walk	0.022	0.086	[− 0.157, 0.183]	0.600	1.501	0.666	
Gr–OU	Stand	− 0.078	0.194	[− 0.478, 0.288]	0.336	0.506	1.977	
**Gr–OU**	**Walk**	**0.204**	**0.078**	**[0.052,0.357]**	**0.995**	**189.476**	**0.005**	*
OC–OU	Stand	− 0.187	0.225	[− 0.631, 0.247]	0.203	0.255	3.917	
**OC–OU**	**Walk**	**0.181**	**0.092**	**[0.007,0.365]**	**0.977**	**42.243**	**0.024**	*
IC–IU	Stand	− 0.114	0.239	[− 0.57, 0.383]	0.316	0.462	2.166	
**IC–IU**	**Walk**	**0.199**	**0.107**	**[− 0.025, 0.399]**	**0.966**	**28.197**	**0.035**	*
OC–IC	Stand	− 0.066	0.239	[− 0.541, 0.399]	0.380	0.613	1.632	
**OC–IC**	**Walk**	**− 0.172**	**0.108**	**[− 0.385, 0.04]**	**0.054**	**0.057**	**17.692**	
OU–IU	Stand	0.009	0.244	[− 0.466, 0.483]	0.516	1.067	0.937	
**OU–IU**	**Walk**	**− 0.156**	**0.097**	**[− 0.341, 0.035]**	**0.050**	**0.053**	**18.950**	

Median, standard deviation (SD), 95% credible intervals (CI) of contrasts between posterior estimates, Posterior probability (PP), evidence ratio (ER), ER01 in favour of the directional hypothesis (PP> 0) and ER10 in favour of the altenrative hypothesis (PP < 0), bold = posteior probability exceeds 85%; star = posterior probability exceeds 95%.

#### Integrated skin conductance responses (ISCR)

Table  9 presents contrasts for ISCR (phasic EDA). The green condition elicited higher compared to the indoor and the outdoor uncrowded (both groups), but indoor crowded elicited higher ISCR than the green for the walking group. For the walking group, ISCR was higher in the crowded compared to uncrowded condition (for both indoor and outdoor), and higher in the indoor crowded than the indoor uncrowded. Other contrasts were unclear.

**Table 9:** Contrasts for integrated skin conductance response (ISCR).

Contrast	Group	Median	SD	95% CI	PP	ER01	ER10	Star
Gr–IC	Stand	2.573	2.800	[− 3.041, 7.873]	0.827	4.776	0.209	
**Gr–IC**	**Walk**	**− 6.487**	**1.643**	**[− 9.688, − 3.249]**	**0.000**	**0.000**	**Inf**	*
**Gr–IU**	**Stand**	**3.848**	**2.703**	**[− 1.516, 9.093]**	**0.917**	**11.121**	**0.090**	
**Gr–IU**	**Walk**	**1.910**	**1.659**	**[− 1.467, 5.059]**	**0.878**	**7.205**	**0.139**	
Gr–OC	Stand	1.764	2.746	[− 3.483, 7.365]	0.743	2.891	0.346	
Gr–OC	Walk	− 0.973	1.620	[− 4.168, 2.211]	0.274	0.377	2.653	
**Gr–OU**	**Stand**	**3.458**	**2.751**	**[− 1.896, 8.799]**	**0.897**	**8.721**	**0.115**	
**Gr–OU**	**Walk**	**2.367**	**1.636**	**[− 0.804, 5.701]**	**0.927**	**12.699**	**0.079**	
OC–OU	Stand	1.703	3.258	[− 4.411, 8.252]	0.696	2.294	0.436	
**OC–OU**	**Walk**	**3.360**	**1.909**	**[− 0.382, 7.115]**	**0.960**	**23.691**	**0.042**	*
IC–IU	Stand	1.230	3.272	[− 5.288, 7.513]	0.645	1.819	0.550	
**IC–IU**	**Walk**	**8.399**	**1.918**	**[4.703,12.346]**	**1.000**	**Inf**	**0.000**	*
OC–IC	Stand	0.838	3.290	[− 5.833, 7.035]	0.600	1.502	0.666	
**OC–IC**	**Walk**	**− 5.535**	**1.912**	**[− 9.408, − 1.898]**	**0.003**	**0.003**	**319.000**	*
OU–IU	Stand	0.363	3.250	[− 6.132, 6.684]	0.543	1.189	0.841	
OU–IU	Walk	− 0.478	1.923	[− 4.222, 3.397]	0.400	0.666	1.502	

Median, standard deviation (SD), 95% credible intervals (CI) of contrasts between posterior estimates, Posterior probability (PP), evidence ratio (ER), ER01 in favour of the directional hypothesis (PP > 0) and ER10 in favour of the altenrative hypothesis (PP < 0), bold = posteior probability exceeds 85%; star = posterior probability exceeds 95%.

## Discussion

This study investigated the psychological responses resulting from the exposure of people to different types of urban environments (urban green, urban outdoor, urban indoor), under different levels of social density (crowded vs uncrowded), using electrophysiological (mobile EEG) and psychophysiological (mobile EDA) measures. Our secondary aim was to investigate in a controlled environment how active walking, compared to standing, impacts the subjective experience of individuals as well as their neurophysiological measures. 42 participants were asked to walk or stand on a treadmill while watching six consecutive videos of different environments and self-reported their emotional state immediately after each exposure. We analysed the EEG signal to assess cognitive and attentional control measuring the theta/beta ratio (TBR)^101,102^, executive functioning by measuring increase in frontal-midline theta (FMT)^103,104^, attention restoration by measuring frontal alpha (FA)^9^, and positive affect/approach motivation by measuring frontal alpha asymmetry (FAA)^105,106^.

Our results support current theories on the psychological effects of different types of environments. We show that in a pedestrian context, experiencing a space in higher social density leads to higher self-reported and physiological arousal, and increased cognitive and attentional demands (higher theta/beta ratio). However, although we observed more negative self-reported valence in crowded environments, electrophysiological measures suggest more positive responses to the environments in crowded conditions. Together these findings encourage additional research into more types of urban environments beyond the nature-urban dichotomy^13^, and into the influence of different levels of human activity^9^. Below, we first discuss the effects of environmental type (green, indoor, and outdoor) followed by the effects of crowding on the experience of space, finally we discuss the role of walking in the measurement of emotional responses to the environment, present the limitations of this study and future work.

In line with ample research on the restorative effects of nature^22,26,27^, we found that participants reported more positive emotions and lower arousal after watching videos of the green space compared to either urban indoor or urban outdoor spaces. Green spaces evoked lower TBR compared to the crowded videos, but higher TBR compared to the uncrowded, implying that green spaces evoked more mind-wandering and crowded scenes led to less attentional control^101^. Higher frontal alpha power, suggesting attention restoration^11,24,27^, was observed for the standing group, but evidence was unclear for the walking group. Analyses of frontal alpha asymmetry reveal an interaction with physical activity, as the standing group produced higher FAA (associated with *approach* motivation) towards the green space compared to the indoor space, but not the outdoor space; for the walking group the crowded outdoor space resulted in higher FAA. This suggests that the either physical activity or providing a context of walking may shift the affective processing of environments. Note that we did not directly measure whether crowding influences the restorative effects of the natural environment^9^. However, the walking group responded more positively to the urban outdoor scene. This could be explained by individuals’ environmental preferences for urban spaces^107^, the restorative effects of sky view^108^, or by the positive effects of urban design^16^.

Second, regarding the influence of crowds, our results confirm the hypothesis that crowded scenes will evoke higher arousal and lower valence^52,58^. In addition, our analysis of brain activity revealed higher TBR in the crowded environments, a measure associated with reduced attentional control^101,102^, and also higher physiological arousal (ISCR and SCL). Overall, these findings are in line with earlier theories about the ‘cognitive complexity’ associated with crowding^54,109^, as well with behavioural studies suggesting that even moderate artificial crowds in a virtual scene can have a ‘distractor effect’ recruiting more attentional resources^110^. Notably in our study, although participants reported more negative valence after watching the crowded scenes, analysis of frontal alpha asymmetry (FAA) pointed to a more complex reaction to crowds. For the outdoor environment, the crowded scenes elicited higher FAA than non-crowded scenes for the walking group, but lower FAA for the standing group. The reverse was observed for the indoor environment, where the walking group had higher FAA in the uncrowded condition, while the standing group had higher FAA in the crowded condition. FAA is considered an index positive and approach emotions^106,111–113^. Thus our results suggest the walking group experienced a more positive reaction to the outdoor environment when it was more crowded, but less positive in the case of the indoor environment, whereas the opposite pattern was observed for the standing group. This suggests treadmill walking influenced the allocation of attention^97^, as well as the affective processing of the scenes. Given that we did not observe a similar difference in self-reported emotions, this implies that walking may have pre-cognitive effects on affective processing of environments. This mismatch between the immediate experience and the post-stimulus appraisal of crowded environments, is in line with the ambivalent appraisals towards crowds which is sometimes negative^58,114^, and other times positive^60,61^. In line with previous studies, an alternative explanation could be that people subconsciously require less peri-personal space in outdoor compared to indoor settings^68^. Further research with more stimuli is needed to elucidate this interaction between space typology and the experience of crowds.

Third, we found that exposure to an outdoor urban space produces more positive and calm emotional responses than indoor space at either level of crowding. We observed higher (i.e. more positive) FAA), and lower psychophysiological arousal (SCL and ISCR). The EEG data suggest that exposure to the outdoor environment, compared to indoor, was associated with improved executive functioning (higher FMT and lower TBR)^101,103^. This could be attributed to a variety of spatial attributes such as: openness, lower enclosure and view of the sky^108^. For instance, it is worth noting that in the urban outdoor scenes, the viewer has a clear view of the sky and several distinct buildings; in contrast, the indoor walkway has also long vistas but limited to retail shops. Notably, the paths in the indoor and outdoor videos were filmed on the same street and are practically overlapping, given that the indoor environment is an underground connector two levels below the street-levels, connecting a metro station with an adjacent shopping mall. In other words people in the same locations can choose to either walk above or below ground.

Last, we considered whether walking, as opposed to standing, influence the subjective experience or the psychophysiological measures of emotion? Firstly, our results suggest that participants of the two groups felt equally immersed (feeling of presence) while watching the video walk-throughs. As a method check, we found that although we applied the recent technique of Artifact Subspace Reconstruction (*ASR*) to remove movement and muscle artifacts^86,87^, there was higher signal-to-noise ratio (SNR) in the walking group^115^. Note that SNR values were calculated on cleaned datasets (i.e., after removing rejected artefact ICs). In line with previous work^116^, we found evidence that mild physical activity (i.e. walking) increases physiological arousal (higher SCL), but the difference between groups was attenuated using the more robust measure of ISCR^117^. However,in line with idea that positive affect increases more when people walk^21,32^, we found differences in the effect of different stimuli depending on which group participants were assigned to, for instance in terms of FAA. Perhaps beyond physical activity, the act of walking mitigated the negative effects of crowding, by providing a sense of agency or control over one’s situation, or by evoking a different behavioural context (e.g. associations of visiting these environments, rather than viewing them). These results should be further investigated with additional stimuli. Taken together these findings make a methodological contribution to the rapidly evolving field of environmental neuroscience, especially in bridging laboratory and field studies.

Our approach presents several limitations. In order to minimise the potential influence of fatigue for the walking group, our experimental setup consisted of a small number of stimuli which inevitably limited the environments presented. Considering the attentional demands of treadmill-walking, these are lower than actual walking due to the lack of inertial stimulation^97^, thus more pronounced differences between the groups may be observed in field studies. Cultural factors and individual preferences may have influenced how crowds are appraised. The lack of crowds in the green environments, due to practical constraints, limit our ability to understand how the presence of people influences the experience of nature^9^. Also, due to our experimental design the effect of the walking on the subjective experience and appraisal of environments could only be studied in a between-subjects analysis. Future work could investigate how other types of urban environments and multiple levels of social density interact and influence people’s attention, cognition and subjective experience.

In summary, we conducted laboratory study to understand the joint influence of environment typology (urban outdoor and indoor, natural), crowding and walking influence subjective experience. The results suggest that green environments evoke more positive affect than urban spaces, that outdoor environments are perceived more positive than indoor environment and, finally, uncrowded—compared to crowded—spaces are associated with more calm and positive self-reported emotions, reduce physiological arousal, and lower attentional demands. Thus the present study makes a methodological contribution to the field of environmental neuroscience comparing walking and static experience of environments, and provides new insights into the psychological experience of urban environments.

## Methods

### Participants

In total, 42 participants (20 female; mean age 22.3, range 19–32 years) participated in this study, recruited through advertisements on an online portal for research studies, as well as through serendipitous methods (participant details are reported in Table 10). Recruitment criteria were: age (18–45 years old), normal or corrected-to-normal vision, right-handedness, as well as the ability and willingness to walk on a treadmill for approximately 20 min. The sample size was determined so as to have 20 participants in each group (walking and standing). Two more participants were recruited to compensate for data lost due to technical issues during data recording, however 6 EEG datasets and 8 EDA datasets were further excluded during data analysis; COVID-19 related restrictions hindered additional data collection to compensate this. Data collection for this study was conducted in Singapore. The study was approved by the ETH Zürich Ethics Committee (Project ID: B_EK_N-164-2019). All methods described in this section were performed in accordance with the Declaration of Helsinki, and with the relevant guidelines and regulations.

**Table 10:** Participant characteristics.

	Total	Group		Test	P value
		Standing	Walking		
	n = 42	n = 20	n = 22		
Gender				Chi Square: 0	0.988
Female	20 (47.6%)	9 (45%)	11 (50%)		
Male	22 (52.4%)	11 (55%)	11 (50%)		
Age				T-Test: 0.87	0.391
	22.29 (2.23)	22.60 (2.56)	22.00 (1.90)		
Height				T-Test: 0.53	0.597
	167.45 (9.17)	168.25 (9.00)	166.73 (9.46)		

### Materials

**Figure 3:**
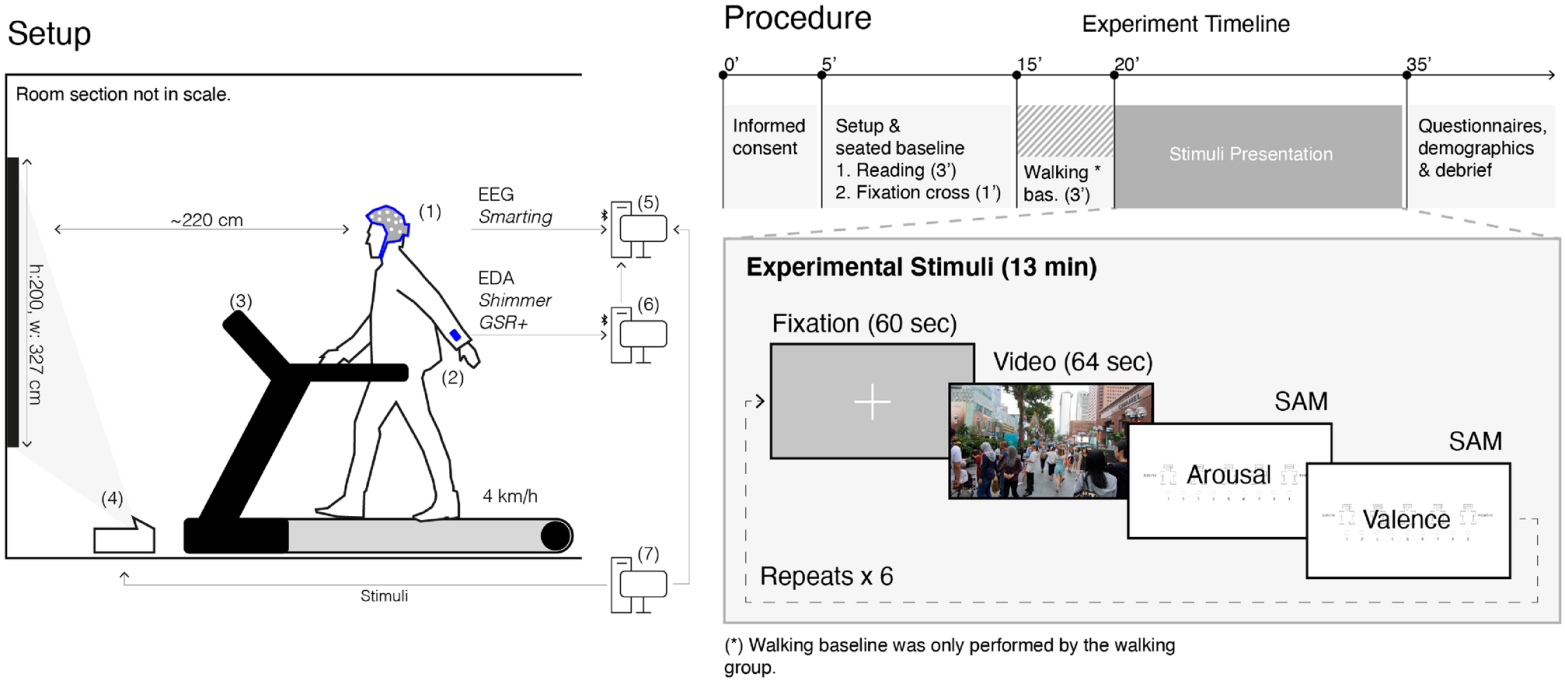
(**A**) In our experimental setup, participants walked (fixed 4.0 km/h speed) or stood on a treadmill (i.e. exactly at the same position), while watching video walk-throughs on a wall-projection. Data from the stimuli presentation, and the EDA sensors were synchronised and recorded together with the EEG data using the Lab-Streaming Layer framework. (**B**) Diagram of the experiment procedures.

Figure 3 illustrates the experimental setup (A) and procedure (B). The experiment consisted of a 2 $\times$ 5 factorial design with *group* (walking or standing) as between-subjects factor, and environmental stimulus as within-subjects factor with 5 levels: green (Gr), indoor crowded (IC), outdoor crowded (OC), and indoor uncrowded (IC), and outdoor uncrowded (OC). Each video lasted for 64 s, including 2 s fade-in and 2 s fade-out. The choice for the number of videos and their duration was to emulating the actual experience of walking through an environment for approximately 75–100 m, or 1 min, which is a typical street block size.The inter-stimulus interval was set to the same duration to serve as a baseline (pre/post) for analaysis. A second important aspect was limiting the total duration of the main phase of the experiment to 15 min so that participants do not experience fatigue from standing or walking. Our primary objective was to examine the interaction between crowding and environment. Consequently, due to short duration of the experiment, and the resulting small number of stimuli, we opted for a between-subjects comparison with respect to the effect of walking.

**Figure 4:**
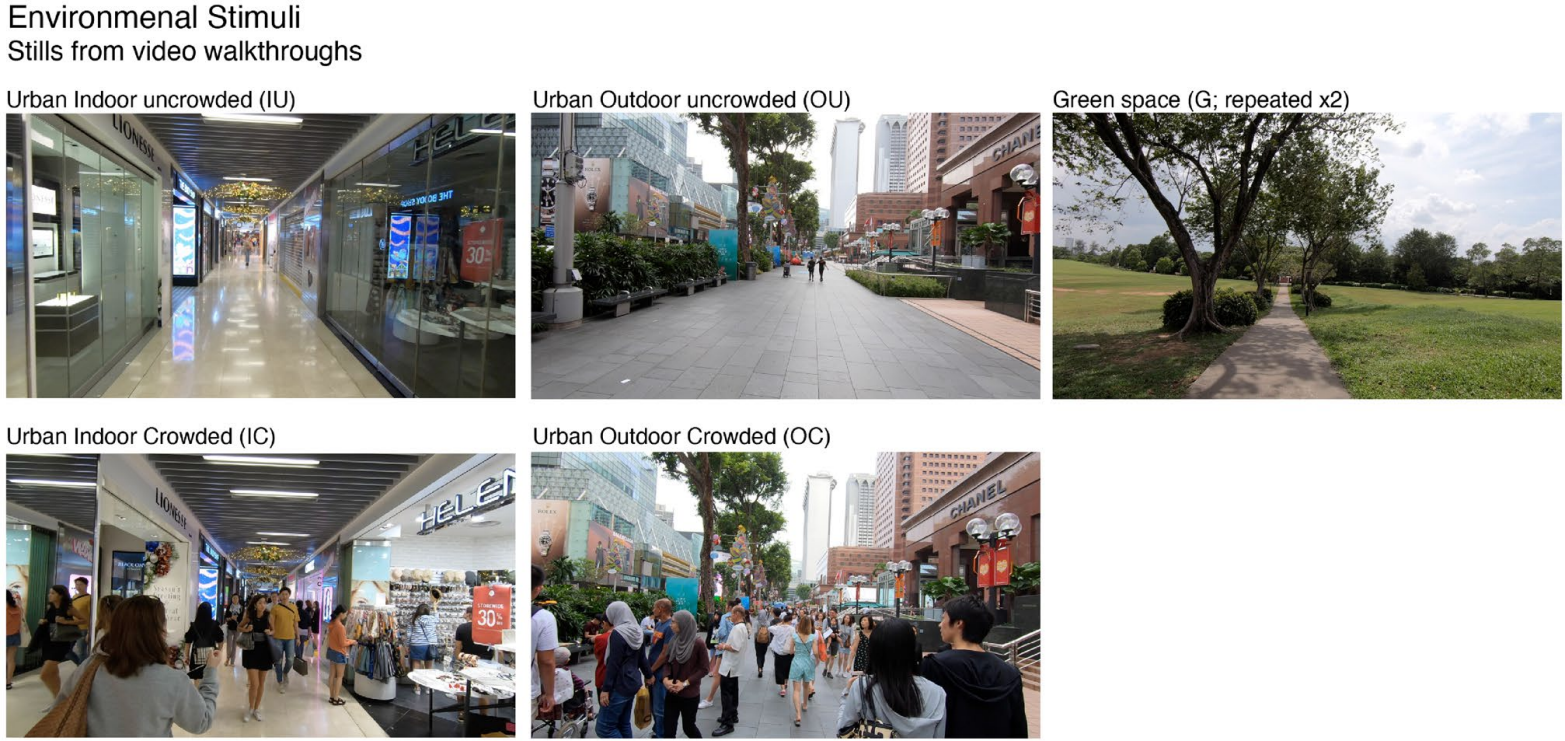
Participants watched 5 video walk-throughs of different environments while walking or standing (between subjects), immediate post-stimulus emotional response using the self-assessment manikin.

Environmental exposure was achieved through the presentation of five (5) first-person video walk-throughs, one for each condition (Fig. 4). The stimuli were created to have comparable characteristics in terms of a single, linear walkway without any turns in the direction of movement, and with no visible end-boundary. All videos were filmed in Singapore. Therefore, the overall environmental conditions were familiar to participants.The green space is located in the entrance of the Chinese Garden in Jurong East. The outdoor space is along Orchard Road (Singapore’s major retail boulevard) and the indoor space was filmed at the retail-lined underground corridor connecting a Mass Rapid Transit station (MRT, Singapore’s metro) with two adjacent major shopping malls. Specifically, the urban indoor location (IC/IU) is an underground walkway (passage) running exactly underneath the location of the urban outdoor (OC/OU). They were chosen precisely because they both reach very high levels of pedestrian crowd during rush-hour, and indeed because they are alternative routes—daily people chose to walk through one or the other raising the question about the psychological effects of this choice. The videos were filmed in the early morning and early afternoon in order to maximise the differences in the pedestrian crowd flows.

The environments presented contain certain limitations. The walkway width, views to the sky, and boundary differ. Further, due to practical limitations it was not possible to film a linear walkway in a green space with matching low and high crowds, thus the green environment does not include any people. The urban environments were presented twice (once with high and once with low levels of pedestrians), the green space was also presented twice and to account for potential presentation effects/repeated measure, we report separately the first and the second time it appeared for each participant (i.e. G1 and G2 respectively).

### Apparatus

Videos were projected onto a white wall (projection width 327 cm and height 220 cm) using an Optoma Ultra Short Throw projector (EH320UST; resolution: 1920 $\times$ 1080 pixels; Optoma Corporation, New Taipei City, Taiwan). Participants from both groups were positioned at the exact same location on a treadmill (AIBI AB-T958; dimensions: 181 (L) $\times$ 85 (W) $\times$ 128 (H) cm). For the standing group the treadmill was turned off, while for the walking group the speed was set at 4km/h and held constant throughout the experiment. Participant position was approximately 220 cm from the wall, which allowed for a full view of the projection, in order to achieve a sense of immersion without requiring head rotation to reduce motion artifacts. Stimulus presentation was implemented as a custom script written in Matlab (R2019b, The MathWorks Inc., USA), using Psychtoolbox^119^. EEG data were acquired at 500 Hz sampling rate using the Smarting wireless mobile EEG, (mBrainTrain, Serbia; EasyCap GmbH, Germany). 24 electrodes were placed according to the 10–20 standard system (FP1, FP2, FPz (DRL), F7, F8, Fz, FC1, FC2, FCz (CMS), Cz, C3, C4, T7, T8, CPz, CP1, CP2, CP5, CP6, TP9, TP10, Pz, P3, P4, O1, and O2). Abrasive electrolyte gel (Abralyt HiCl, Easycap GmbH, Germany) was applied under each electrode to ensure skin impedance values below 10 kOhm. EDA was recorded using the mobile device Shimmer GSR+ (Shimmer Realtime Technologies Ltd, Ireland). Pre-gelled, disposable snap Ag/AgCl electrodes (EL507, Biopac Systems Inc., Santa Barbara, CA, United States) were placed at the intermediate phalanges of the index and the middle finger of participants’ non-dominant (in all cases, left) hand. The signal amplifier was attached to the lower arm of the participants, above the wrist and secured with an elastic band.

#### Data synchronisation

Presentation of stimuli and data recordings were synchronised using the Lab Streaming Layer framework, or LSL^118^. The LSL LabRecorder (version 1.12) received four inputs: (1) EEG data stream, (2) EDA data streams, (3) keyboard presses, and (4) event markers from the stimulus presentation. EEG data were wirelessly transmitted from the Smarting amplifier, received in the computer using the Bluetooth manager BlueSoleil, and then sent to LSL by the SMARTING Streamer. Experimental stimuli, including instruction text, fixation cross, videos, and SAM were presented using the MATLAB plugin Psychtoolbox^119–121^ and an event marker was dispatched to LSL at the start and at the end of each event. EDA was recorded on the Consensys Pro (Shimmer’s native platform). A timestamped marker was sent to LabRecorder when the EDA recording started.

### Procedure

Upon arrival to the laboratory, participants provided informed consent, the researcher attached the EEG headset and the EDA sensors, and initiated data recording.

#### Baseline phase

A 3-min baseline for EEG and EDA recordings was obtained while participants read a short story on a computer screen (passage from “The Little Prince”; Saint-Exupéry, 2018, Ch. 1, p. 9–16), while sitting comfortably on a chair in a darkened room. Further, to obtain artifact- and task-free baselines for the EEG and EDA signals, a second 1-min baseline was obtained while participants looked at a fixation cross in the centre of a computer screen while sitting calmly in their chairs. Afterwards, participants were asked to step on the treadmill.

All participants were instructed to minimise head movements and relax their facial muscles throughout the experiment to reduce motion and muscle artefacts in the EEG data. Out of precaution, they were instructed to hold the right-sided handle of the treadmill with their right (dominant) hand at all times. Thus, the non-dominant hand (where EDA was measured) could move naturally in order to observe the effects of walking on the EDA signal. For the walking group only a third baseline was recorded while they walked looking at a white fixation cross in the centre of the screen for 3 min.

#### Main phase

The main experiment consisted of the presentation of 6 videos (1 x IC, 1 $\times$ OC, 1 $\times$ IU, 1 $\times$ OU, and 2 $\times$ GS) while participants walked (walking group) or stood (standing group) on the treadmill. Each video was preceded by a 60-s fixation cross in the centre of the screen. After each video, participants were instructed to self-report their emotional state using the Self-Assessment Manikin (SAM)^100^, on a scale from 1 to 9 (9 corresponding to calm in the arousal scale and negative in the valence scale). Because walking on the treadmill makes keyboard use difficult and potentially unsafe, participants from both groups indicated their response verbally (i.e speaking aloud the number) and the researcher entered the response on keyboard (response time was not recorded). To control for order effects, videos were presented counterbalanced in the sequence GS–IC–OU–GS–OC–IU, or its reverse, and participants were randomly assigned to either one.

After watching all 6 videos, participants stepped down from the treadmill and returned to the computer to complete a set of questionnaires, including a 7-point Likert scale about their general attitude towards crowded spaces (“How much do you like to walk in crowded environments?”), the MEC-Spatial Presence Questionnaire, a standardised questionnaire about their sense of present while watching the scenes(MEC-SPQ)^122^, and general demographics (age, gender, height). As we were mainly interested in the spatial dimension of the perceived presence, we only analysed the Spatial Presence: Self Location (SPSL) subscale consisting of 8 items, such as “I felt like I was a part of the environment in the presentation”.

### Data preprocessing

All data and event marker streams were automatically synchronised in post-processing using the LSL function (xdf_load).

#### EEG

EEG data were preprocessed offline using custom Matlab scripts and EEGLAB toolbox (Version 2019.1)^123,124^. Continuous EEG data from each participant were band-pass filtered (1–40 Hz), line noise was removed (*pop_cleanline* function), and then artifacts and bad electrodes were automatically detected and removed or attenuated using the Artifact Subspace Reconstruction (ASR; *clean_rawdata* function)^86^. We then performed independent component analysis (ICA) using multiple models with shared components (*runamica15* function) on re-referenced-to-average data. From these components, single equivalent dipoles were estimated (*pop_multifit* function), symmetrically constrained bilateral dipoles were detected, and independent components were rejected (*ICLabel* function, residual variance < 15).

We found a difference in the number of rejected ICs between the two groups. In particular, there were fewer rejected ICs for the standing group (M = 3.7, SD = 0.43) compared to the walking group (M = 7.3, SD = 0.51) and the difference was statistically significant ($t(33)=-5.414, p<0.001, r = 0.686, Cohen's d = 2.062$).

We compared signal-to-noise ratio (SNR) between the two experimental groups to evaluate possible differences in EEG signal quality between walking and standing *after* preprocessing the signal using ASR and ICA^85–87,125^. SNR was computed for each participant and electrode separately by calculating the percentage of artifacts in a continuous EEG data segment^126^ (Eq. 1):

1 $$\begin{aligned} SNR = 10 * log (\frac{EEG_{{clean}^{2}}}{(EEG_{filtered} - EEG_{clean})^{2}} \end{aligned}$$

Finally, in order to control for individual differences in the overall signal quality, we computed the difference between SNR from the sitting baseline period (60 s) and each of the 6 inter-stimulus intervals, resulting in 6 values for each participant.

To assess the effects of the six videos, the preprocessed EEG data for each participant were epoched into 60-s video presentation segments (from second 2 to 62 after video onset, to remove the fade-in and fade-out periods) and the preceding fixation cross segments (0–60 s after onset of the fixation cross). EEG power spectra were computed for each channel and epoch separately (*spectopo* function using zero-padded data to obtain 0.5 Hz resolution and a window overlap of 250 ms). For all frequency-bands, we then calculated the percentage change of power during video presentation compared to the preceding fixation cross period (Cohen, 2014). The following formula was used for baseline correction at each electrode (Eq. 2):

2 $$\begin{aligned} 100 + 100* ( \frac{Power_{Video}-Power_{ISI}}{Power_{ISI}} ) \end{aligned}$$

Four indices were produced for further analysis using the baseline-corrected EEG frequency power data: *Frontal alpha (FA)* which is associated with attention restoration^11^, *Frontal Midline Theta (FMT)* which is associated with cognitive control^103,104^, *Theta/beta ratio (TBR)* which is associated with cognitive load^101,102^, and *Frontal alpha asymmetry (FAA)*^105,113,127^. FA was computed as the average of the baseline-corrected alpha power (8–12.5 Hz) from frontal electrodes (F3, F4, F7, F8, FP1, FP2, AFz, Fz). FMT was calculated by extracting baseline-corrected theta power (4–7.5 Hz) from frontal midline electrodes Fz and AFz. TBR was computed by averaging baseline-corrected theta (4–7.5 Hz) and beta (13–24.5 Hz) power across all frontal electrodes (F3, F4, F7, F8, FP1, FP2, AFz, Fz), and then TBR was calculated by dividing the average frontal theta power by the average frontal beta power. FAA was derived by subtracting the logarithmic alpha power at electrode F4 (right frontal hemisphere) from F4 (left frontal hemisphere), i.e. F4–F3. Therefore, higher FAA indicates higher relative alpha power in the left hemisphere, which is associated more positive/approach affective states^106,111–113^.

#### EDA

EDA signals were preprocessed using the Matlab plugin Ledalab^117^. The raw skin-conductance (SC) signal was downsampled to 16 Hz, a first-order Butterworth filter with a 5Hz cut-off was applied, and then it was submitted to Continuous Deconvolution Analysis (CDA) set to default optimisation settings. CDA was used to separate the tonic from the phasic components of the signal. We then computed two indicators: the skin conductance levels (SCL) based on the tonic component produced by CDA, the integrated skin conductance response (ISCR) a measure proposed by Benedek and Kaernbach^117^ to better capture phasic activity.

### Statistical analysis

Finally, for each measure of EEG (alpha, FMT, TBR, FAA) and EDA (SCL, ISCR), we computed the difference between each trial (i.e. 60 s of video, exluding the fade-in/out) and the preceding 60-s inter-stimulus interval (ISI). Statistical analysis, tables and plots were performed using the R language for statistical programming (version 4.0.2; R Core Team 2020), using tidyverse, and raincloud plots^128^. Bayesian t-tests were implemented using BayesFactor R-package^129^. The effects of the walking and environmental condition were analyzed using bayesian hierarchical (mixed) models fitted in STAN^130^ access from the R package *brms*^131^. To better handle potential outliers in the data, we implemented heavy-tailed distribution (student, robust regression) while accommodating heterogeneity of variances across groups. Sampling was performed with 4 chains of 4000 iterations (each 2000 burn-in) and convergence was assessed using effective sample size, Rhat < 1.01 and posterior predictive checks (code available in Supplementary Materials). We specified fixed effects for group and condition, and a random intercept term for participants to account for individual differences, and a random intercept for the counterbalancing schedule. The *emmeans* R package (version 1.4.8; Lenth, 2020) was used estimate marginal means (EMM) from the posterior distribution, and then compute planned contrasts. For the interpretation of the resulting Bayes Factors we followed Jeffreys^132^ categorisation as follows: BF 1–3: anecdotal evidence; BF = 3–10: substantial evidence; BF = 10–30: strong evidence; BF = 30–100: very strong evidence; BF> 100: decisive evidence. In the results we denote evidence for the null hypothesis as BF01 and evidence in favour of the alternative hypothesis as BF10; we refer to bayes factors as ‘evidence ratio‘ (ER) for the case of directional hypotheses (e.g. posterior probability> 0)^131^.

## Supplementary Information

Supplementary Information.

## Data Availability

The data that support the findings of this study are available from the corresponding author upon reasonable request.
