# Supplementary material for: A mobile EEG study on the psychophysiological effects of walking and crowding in indoor and outdoor urban environments
Source: Sci Rep. 2022 Nov 2;12:18476. doi: 10.1038/s41598-022-20649-y (PMC9628500; doi:10.1038/s41598-022-20649-y)
Supplement: Supplementary file 1 — Supplementary Information. [file 41598_2022_20649_MOESM1_ESM.pdf]

Effects of Walking and Crowding - BRMS MODELS

This analysis document is accompanying the journal article “Effects of walking and crowding on the subjective experience of indoor and outdoor urban environments: a controlled, mobile neurophysiological study” by Mavros, Waelti, Nazemi, Ong and Hoelscher.

The file includes the statistical analysis, both code and results, and it allows to reproduce the (bayesian) statistical models presented in the paper.

Analysis Plan

In the present document we show the descriptive plots (histograms of the data), the code used to fit the models using the R-package *brms*, the posterior predictive checks of the resulting model (pp_check), and the model results. These were not included in the main manuscript for brevity, but can be useful to assess the models. Model estimates are printed using the *bayestestR* package, which provides descriptive statistics if the estimated parameters (Median, 95% Credible Intervals) and model diagnostics (Rhat and ESS) and probability direction (pd, i.e. the proportion of the posterior estimate that is in the same direction as the median of estimate, and thus the strength of evidence for the presence or absence of an effect).

For all these dependent variables we fit a mixed-effects (hierarchical) model, specifying a random effect for *participant* and for counterbalancing *schedule*, and fixed effects for *condition* (i.e. the stimuli) and for *group* (standing or walking). We fit Bayesian mixed-effects (hierarchical) models using brms to the following dependent measures:

1. Self-reported Valence (measured using the self-assessment manikin, or SAM)
2. Self-reported Arousal (also measured with SAM
3. Frontal midline theta (EEG)
4. Frontal alpha assymetry (EEG)
5. Theta / beta ratio (EEG)
6. Frontal alpha (EEG)
7. Skin conductance levels
8. Skin conductance responses

SETUP

Self_Reported Data

Load data

We have self-reported from all participants (N = 42), so in total (42 * 6) data-points.

```
stats_behavioural_table <- read_csv("_data/self_reported_data.csv")
head(stats_behavioural_table)
```

Condition <chr>	Participant_ID <dbl>	Group <chr>	Arousal <dbl>	Valence <dbl>	Schedule <chr>	LikingCrowds <dbl>
G1	1041	Standing	8	2	First stimulus = G1	2
IC	1041	Standing	3	7	First stimulus = G1	2
OE	1041	Standing	5	4	First stimulus = G1	2
G2	1041	Standing	8	1	First stimulus = G1	2
OC	1041	Standing	3	7	First stimulus = G1	2
IE	1041	Standing	8	7	First stimulus = G1	2

6 rows

```
v <- stats_behavioural_table %>%
  ggplot(aes(Valence)) +
  geom_histogram(outline.type = "upper") +
  facet_wrap(.~Condition) +
  labs(title = "Valence") +
  scale_x_continuous(limits = c(1,9))

a <- stats_behavioural_table %>%
  ggplot(aes(Arousal)) +
  geom_histogram(outline.type = "upper") +
  facet_wrap(.~Condition) +
  labs(title = "Arousal") +
  scale_x_continuous(limits = c(1,9))

v + a
```

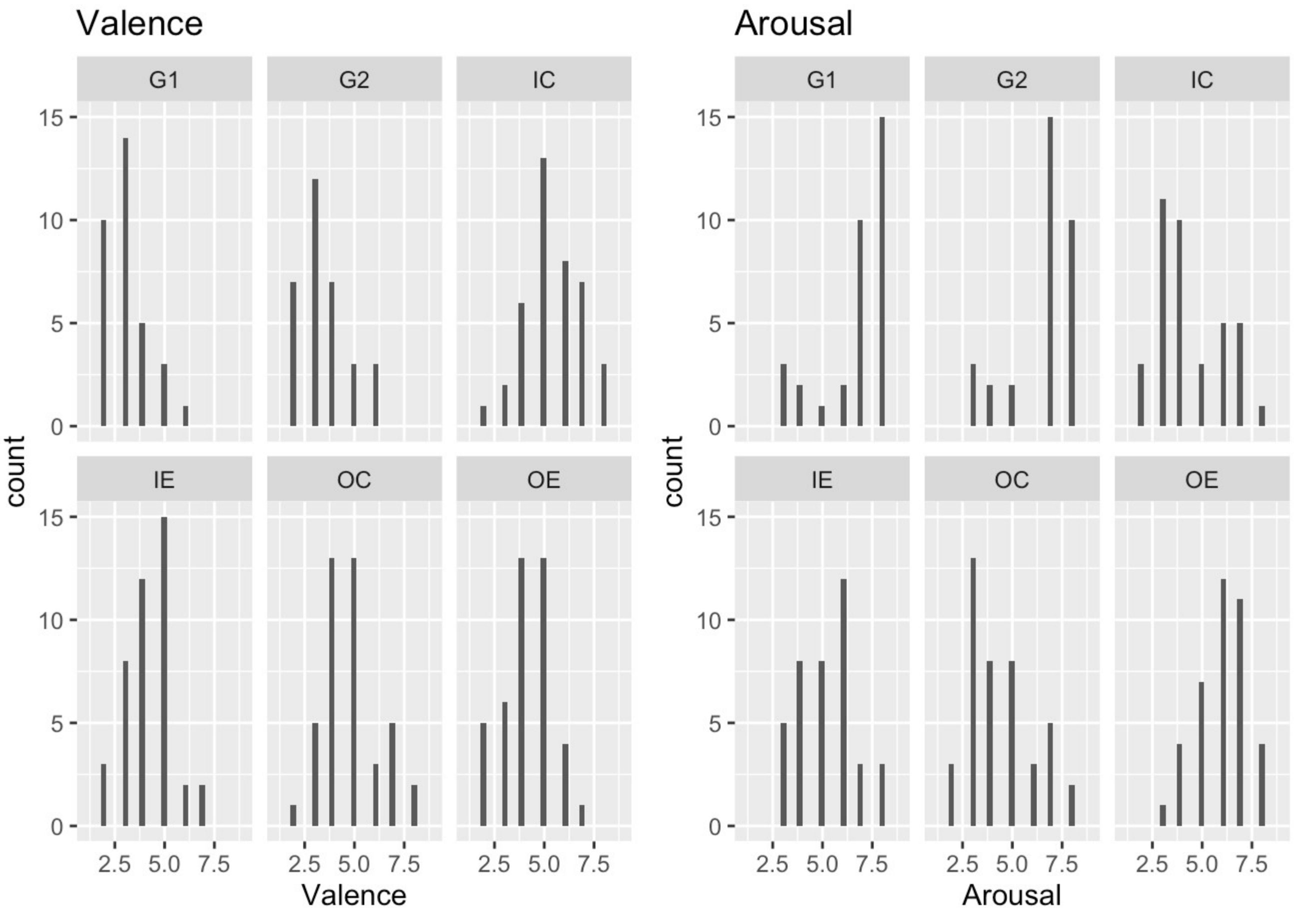

1 Valence

```
bf_sam_valence <- brm(formula = bf(Valence ~ Group * Condition + (1 + Condition | Par
ticipant_ID) + (1|Schedule)),
                          stats_behavioural_table,
                          family = cumulative(),
                          iter = 4000,
                          seed = 4343,
                          prior = set_prior("normal(0, 10)", class = "b"),
                          save_pars = save_pars(all = T),
                          file = here::here("_brmsfiles/bf_sam_valence"),
                          cores = parallel::detectCores(),
                          control = list(adapt_delta = .999, max_treedepth  = 15)
)

pp_check(bf_sam_valence, type = "bars")
```

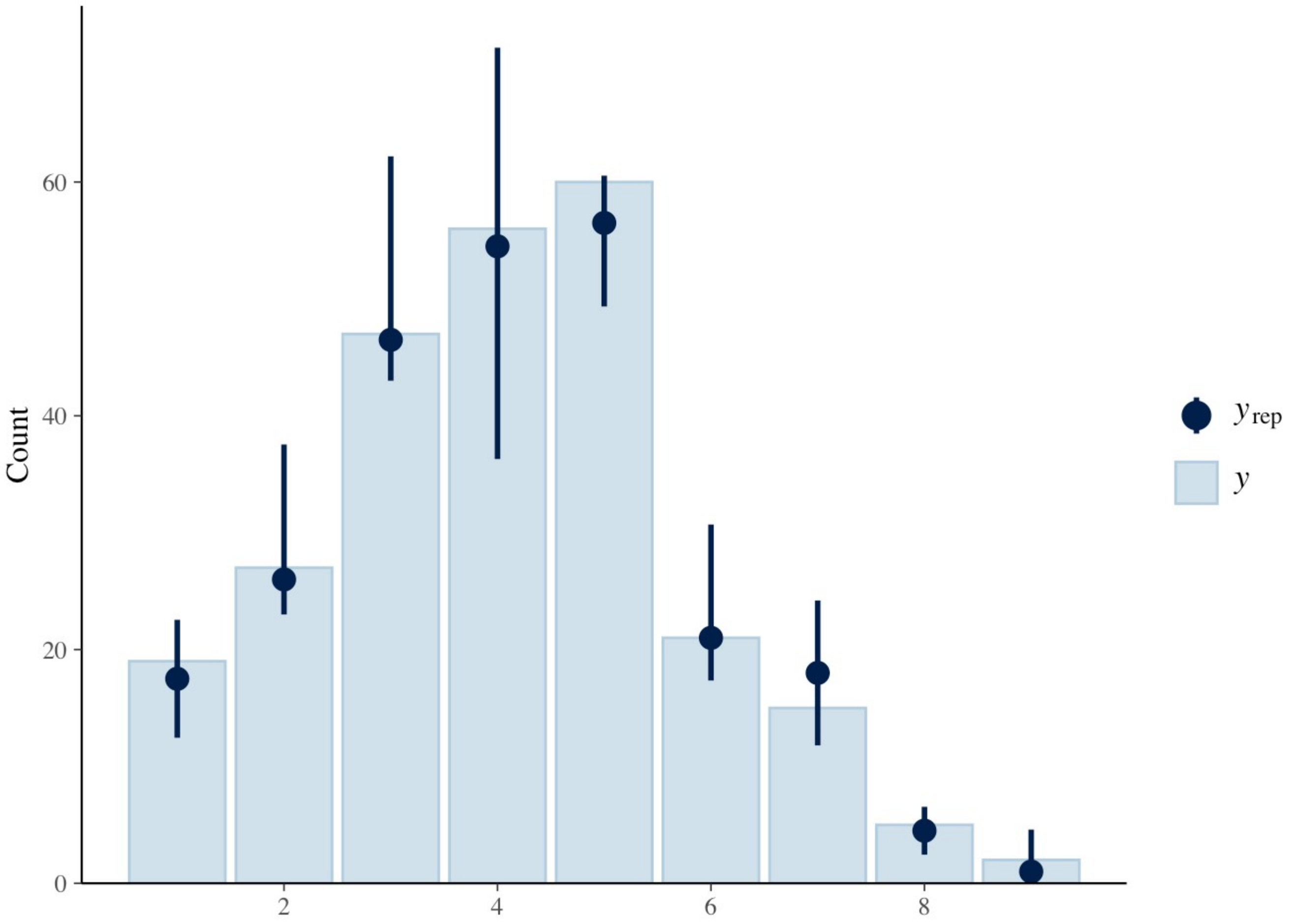


```
describe_posterior(bf_sam_valence, test = "pd")
```

	Parameter <chr>	Median <dbl>	CI <dbl>	CI_low <dbl>	CI_high <dbl>	pd <dbl>	Rh[illegible] <db[illegible]
12	b_Intercept[1]	-3.27913048	0.95	-6.2139347	-1.012269	0.998125	1.0017
13	b_Intercept[2]	-0.99412051	0.95	-3.8324343	1.091305	0.830375	1.0007
14	b_Intercept[3]	1.36588687	0.95	-1.3848743	3.642560	0.851500	1.0012
15	b_Intercept[4]	3.58701991	0.95	0.7565055	6.143204	0.991875	1.0032
16	b_Intercept[5]	6.15706393	0.95	3.0332943	9.068959	0.999750	1.0059
17	b_Intercept[6]	7.67810898	0.95	4.5202248	11.065048	1.000000	1.0075
18	b_Intercept[7]	9.97257337	0.95	6.3435732	14.073116	1.000000	1.0086
19	b_Intercept[8]	12.18343516	0.95	7.9178613	17.159883	1.000000	1.0079
6	b_GroupWalking	-0.09263437	0.95	-1.8071998	1.705332	0.541000	1.0038
1	b_ConditionG2	-0.31857934	0.95	-2.0160170	1.367381	0.649625	1.0003

1-10 of 19 rows Previous **1** 2 Next

2 Arousal

```
bf_sam_arousal <- brm(formula = bf(Arousal ~ Group * Condition + (1 + Condition | Par
ticipant_ID) + (1|Schedule)),
                          stats_behavioural_table,
                          family = cumulative(),
                          iter = 4000,
                          seed = 4343,
                          prior = set_prior("normal(0, 5)", class = "b"),
                          save_pars = save_pars(all = T),
                          file = here::here("_brmsfiles/bf_sam_arousal"),
                          cores = parallel::detectCores(),
                          control = list(adapt_delta = .999, max_treedepth  = 15)
)

pp_check(bf_sam_arousal, type = "bars")
```

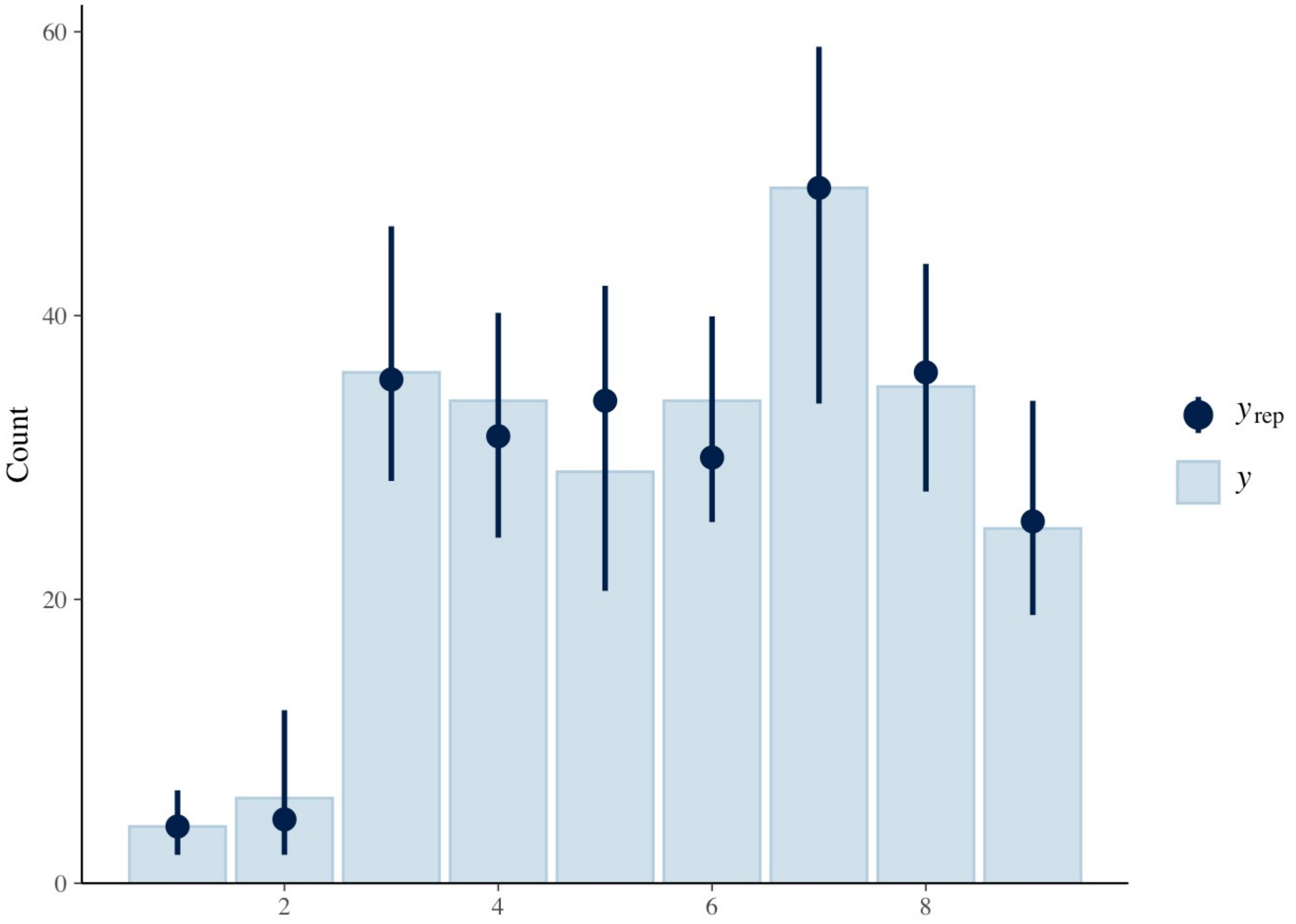


```
describe_posterior(bf_sam_arousal, test = "pd")
```

	Parameter	Median	CI	CI_low	CI_high	pd	
	<chr>	<dbl>	<dbl>	<dbl>	<dbl>	<dbl>	
12	b_Intercept[1]	-11.3957664	0.95	-14.9713141	-8.1489982	1.000000	1.0[illegible]

	Parameter	Median	CI	CI_low	CI_high	pd	
	<chr>	<dbl>	<dbl>	<dbl>	<dbl>	<dbl>	
13	b_Intercept[2]	-9.5060458	0.95	-12.7462519	-6.6438302	1.000000	1.0[illegible]
14	b_Intercept[3]	-6.0462081	0.95	-8.7310409	-3.6582738	1.000000	1.0[illegible]
15	b_Intercept[4]	-4.2511971	0.95	-6.5264767	-1.7147265	0.998625	1.0[illegible]
16	b_Intercept[5]	-2.8577342	0.95	-4.9285544	-0.3268421	0.982875	1.0[illegible]
17	b_Intercept[6]	-1.3202732	0.95	-3.5008791	1.0590898	0.865500	1.0[illegible]
18	b_Intercept[7]	1.0205147	0.95	-1.0287852	3.4846205	0.841375	1.0[illegible]
19	b_Intercept[8]	3.4883818	0.95	1.3094895	6.1055472	0.999500	1.0[illegible]
6	b_GroupWalking	1.3471508	0.95	-0.8039706	3.3195924	0.892625	1.0[illegible]
1	b_ConditionG2	0.4027134	0.95	-1.0835691	1.8377843	0.710000	1.0[illegible]

1-10 of 19 rows Previous **1** 2 Next

EEG DATA

Load data

```
eeg_markers_segments_60s <- readr::read_rds("_data/original_segment4_60s.rds")
eeg_markers_segments_60s %>%
  head()
```

Group	Condition	Participant_ID	Segm…	TBR	FAA	FMT
<fct>	<fct>	<chr>	<dbl>	<dbl>	<dbl>	<dbl>
Standing	Green_1	1041	4	1.4680312	-0.14437121	118.22645
Standing	Green_2	1041	4	1.4803180	0.10489069	91.52706
Standing	Indoor_Crowded	1041	4	1.7005612	-0.04945622	116.57264
Standing	Indoor_Uncrowded	1041	4	1.0960640	-0.02302966	106.88314
Standing	Outdoor_Crowded	1041	4	0.9219474	-0.09625504	90.28144
Standing	Outdoor_Uncrowded	1041	4	0.7098122	-0.15188811	105.82250

6 rows | 1-9 of 21 columns

Every participant has 6 rows, corresponding to the 6 video-stimuli.

```
table(eeg_markers_segments_60s$Participant_ID)
```

```
##
## 1041 1164 1302 1370 1755 1874 2405 2785 2894 3111 3289 4216 4308 4754 4900 5099
##    6    6    6    6    6    6    6    6    6    6    6    6    6    6    6    6
## 5515 5541 5668 5748 5800 5932 6043 6373 6461 6803 6810 7338 7430 7793 8551 8806
##    6    6    6    6    6    6    6    6    6    6    6    6    6    6    6    6
## 8966 9032 9948
##    6    6    6
```

There are EEG data for 35 participants. Sanity check:

```
length(eeg_markers_segments_60s$Participant_ID) / 6
```

```
## [1] 35
```

3 FMT (Frontal Midline Theta)

```
hist(scale(eeg_markers_segments_60s$FMT))
```

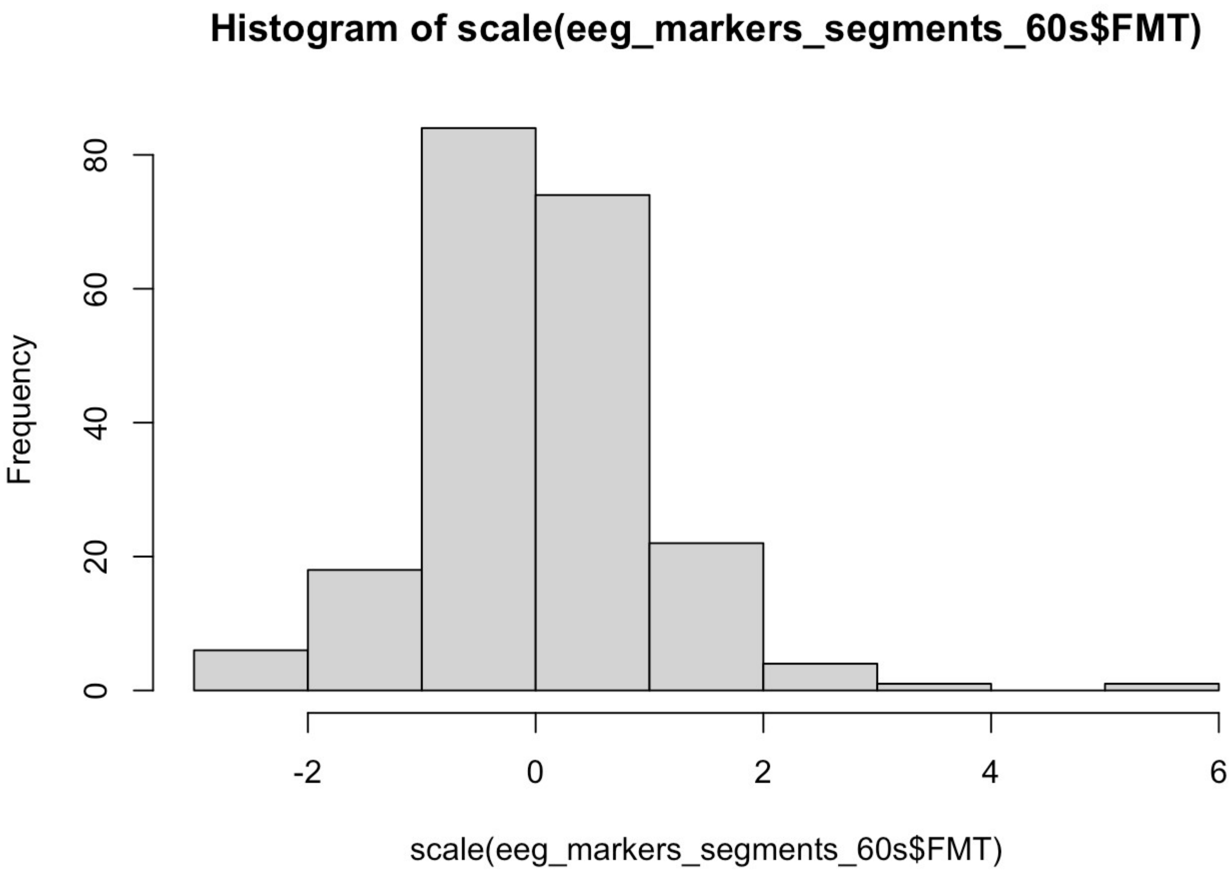

We see that there are`nrow(eeg_markers_segments_60s) - nrow(eeg_markers_segments_60s %>% mutate(FMT_s = scale(FMT)) %>% filter(FMT_s > -4 & FMT_s < 4))` observations, that are more than 4 standard deviations from the mean. We treat this is as an outlier and remove it from the analysis.

```
bf_fmt_int_student_60s <- brm(formula = bf(FMT ~ Group*Condition + (1 + Condition |Pa
rticipant_ID) + (1|schedule), sigma~Group),
                              eeg_markers_segments_60s %>%
                                mutate(FMT_s = scale(FMT)) %>%
                                filter(FMT_s > -4 & FMT_s < 4), # remove one outliers
                          family = student(),
                          iter = 4000,
                          seed = 4343,
                          prior = set_prior("normal(0, 5)"),
                          save_pars = save_pars(all = T),
                          file = here::here("_brmsfiles/bf_fmt_int_student_60s"),
                          cores = parallel::detectCores(),
                          control = list(adapt_delta = .999)
)

pp_check(bf_fmt_int_student_60s)
```

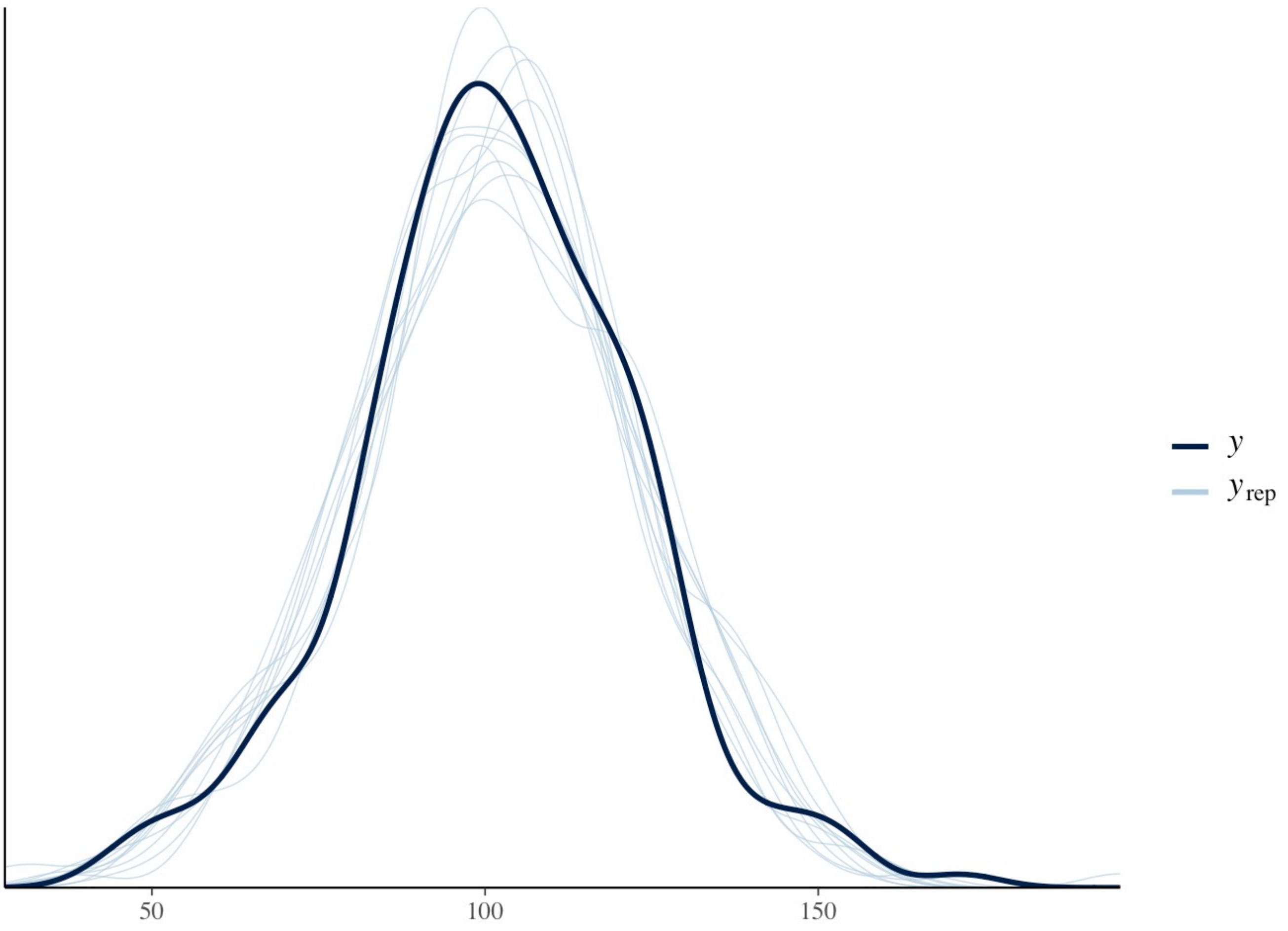


```
describe_posterior(bf_fmt_int_student_60s, test = "pd")
```

	Parameter	Median	CI	CI_low	
	<chr>	<dbl>	<dbl>	<dbl>	
12	b_Intercept	102.50660561	0.95	85.5599181	118.[illegible]

	Parameter	Median	CI	CI_low	
	<chr>	<dbl>	<dbl>	<dbl>	
14	b_sigma_Intercept	2.42327430	0.95	2.1589851	2.[illegible]
6	b_GroupStanding	-1.26289683	0.95	-8.7429459	6.[illegible]
1	b_ConditionGreen_2	-1.48694594	0.95	-6.8491225	3.[illegible]
2	b_ConditionIndoor_Crowded	1.46979101	0.95	-4.0010190	6.[illegible]
3	b_ConditionIndoor_Uncrowded	-1.29746159	0.95	-6.3405907	4.[illegible]
4	b_ConditionOutdoor_Crowded	2.95181101	0.95	-2.6344891	8.[illegible]
5	b_ConditionOutdoor_Uncrowded	1.24655655	0.95	-4.2254586	6.[illegible]
7	b_GroupStanding:ConditionGreen_2	-3.00027911	0.95	-9.2713284	3.[illegible]
8	b_GroupStanding:ConditionIndoor_Crowded	0.38820635	0.95	-6.4780861	6.[illegible]

1-10 of 14 rows | 1-7 of 9 columns Previous **1** 2 Next

4 Frontal Alpha

```
# check for severe outliers
# looks ok
hist(scale(eeg_markers_segments_60s$Alpha_Frontal))
```

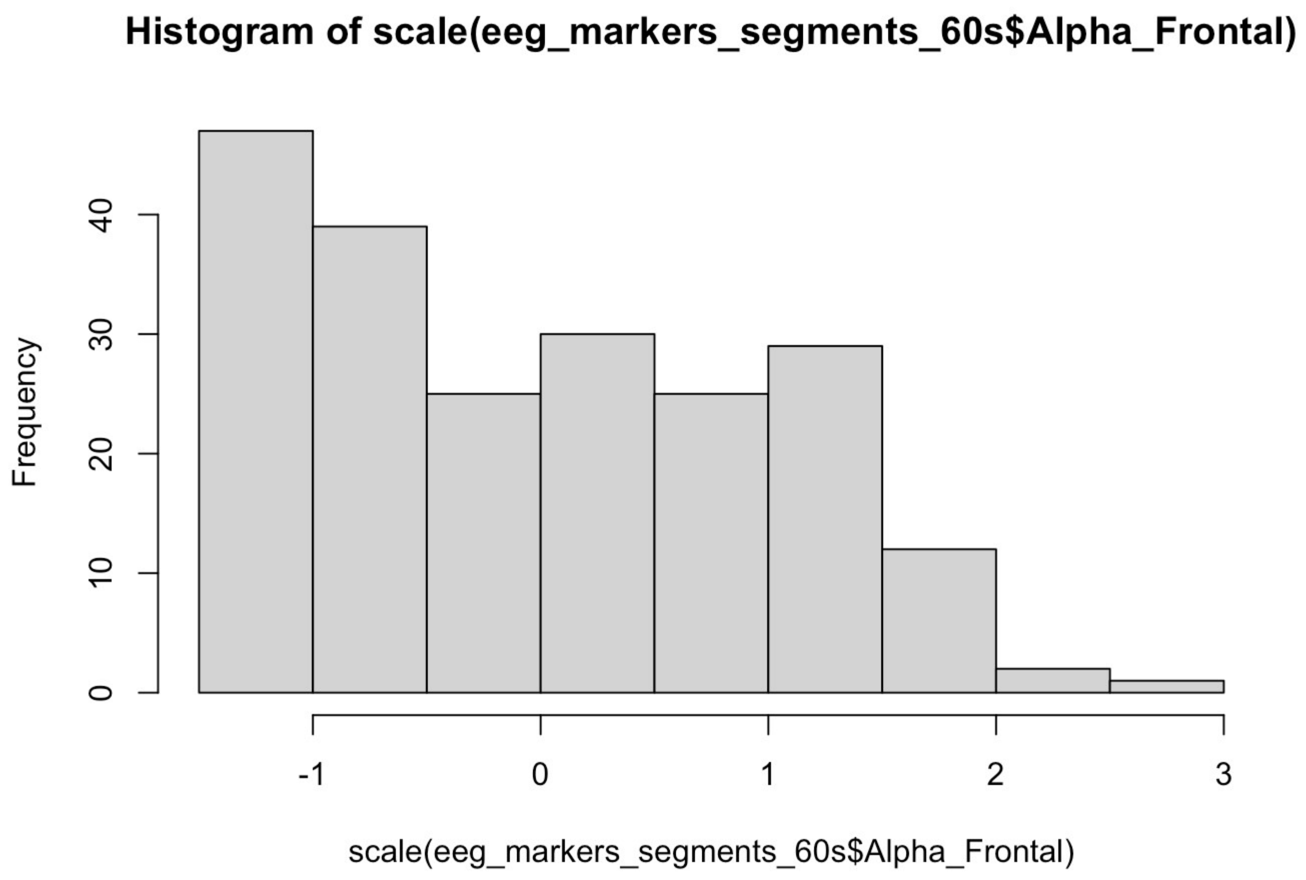


```
eeg_markers_segments_60s %>%
  ggplot(aes(Alpha_Frontal)) +
  geom_density(outline.type = "upper") +
  facet_wrap(.~Group*Condition)
```

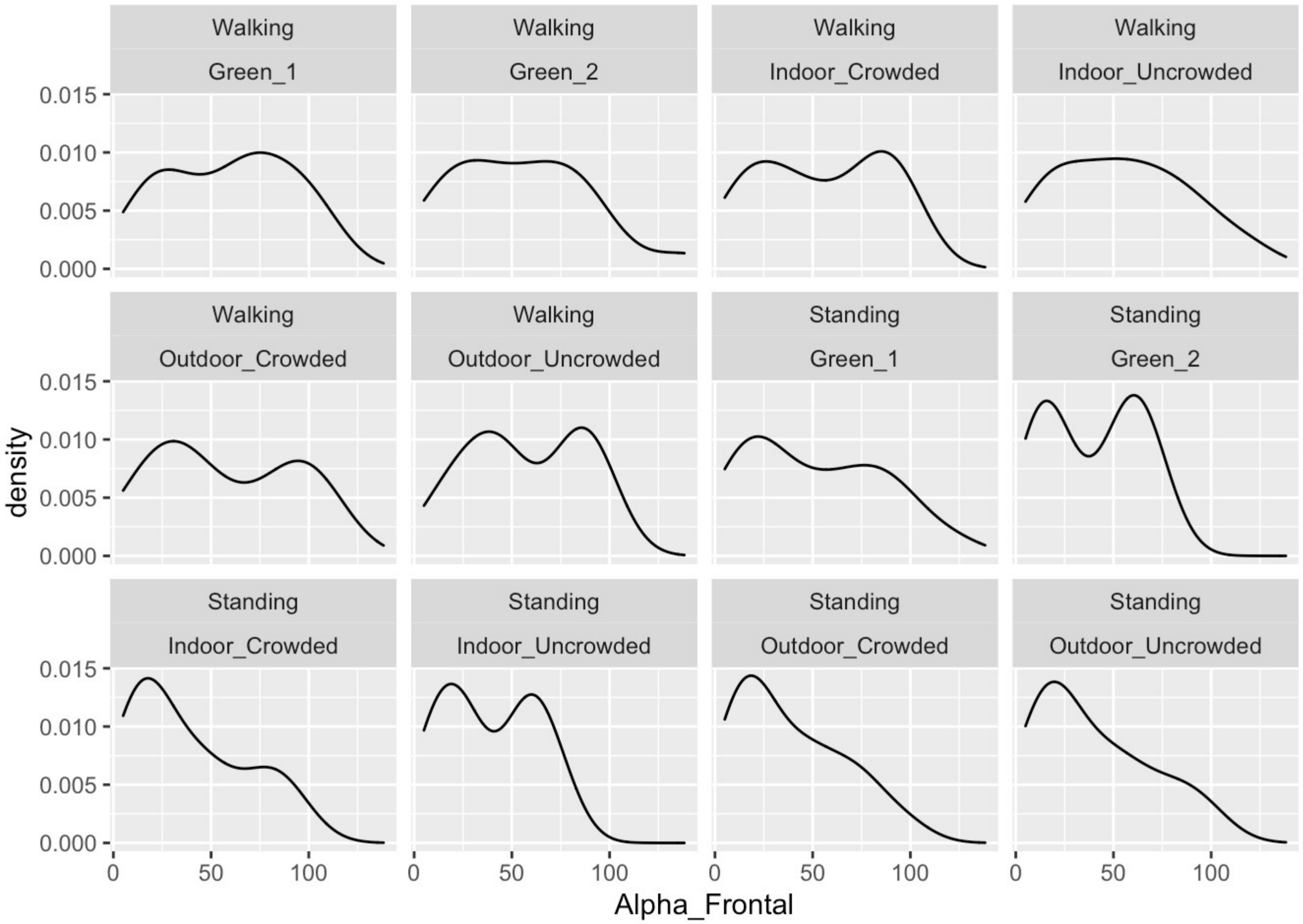


```
bf_alphaFrontal_int_exgaussian_60s <- brm(formula = bf(Alpha_Frontal ~ Group * Condit
ion + (1 + Condition | Participant_ID) + (1|schedule), sigma ~ Group),
                              eeg_markers_segments_60s, #
                          family = exgaussian(),
                          iter = 4000,
                          seed = 4343,
                          cores = parallel::detectCores(),
                          prior = set_prior("normal(0,5)"),
                          save_pars = save_pars(all = T),
                          file = here::here("_brmsfiles/bf_alphaFrontal_int_exgaussia
n_60s_n0-5")
)

pp_check(bf_alphaFrontal_int_exgaussian_60s)
```

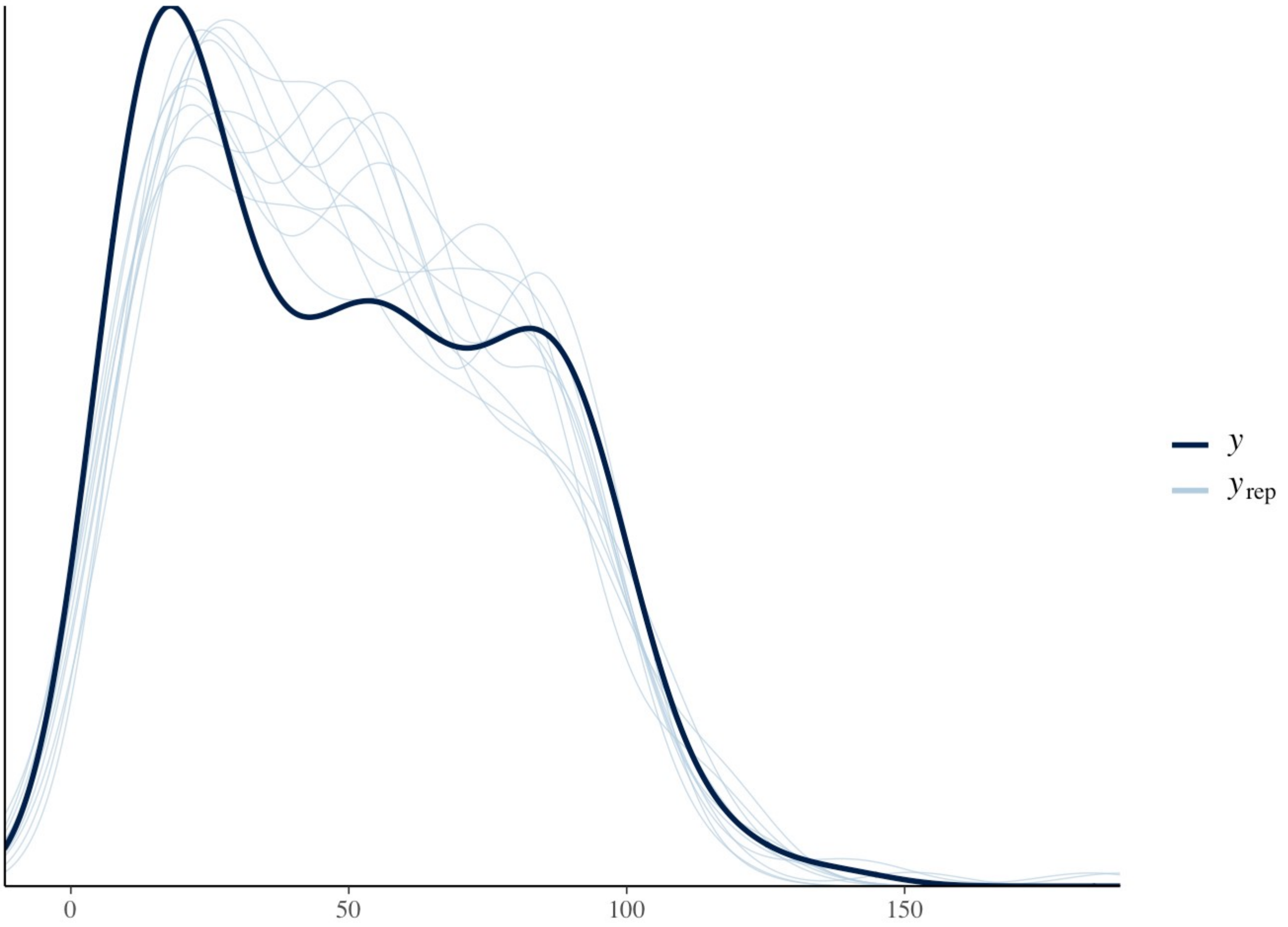


```
describe_posterior(bf_alphaFrontal_int_exgaussian_60s, test = "pd")
```

	Parameter <chr>	Median <dbl>	CI <dbl>	CI_low <dbl>	CI_h[illegible] <[illegible]
12	b_Intercept	54.8962873	0.95	34.575703	106.3245
14	b_sigma_Intercept	1.1361119	0.95	-3.251961	1.9782
6	b_GroupStanding	-3.5349270	0.95	-11.035264	5.3660
1	b_ConditionGreen_2	-4.3050589	0.95	-7.663973	-0.1997
2	b_ConditionIndoor_Crowded	-3.1235770	0.95	-6.696863	1.2939
3	b_ConditionIndoor_Uncrowded	-4.2245386	0.95	-8.553523	0.2500
4	b_ConditionOutdoor_Crowded	-2.2266581	0.95	-6.387896	2.8352
5	b_ConditionOutdoor_Uncrowded	-2.1126586	0.95	-5.549295	2.5657
7	b_GroupStanding:ConditionGreen_2	-0.4992405	0.95	-5.008784	3.9793
8	b_GroupStanding:ConditionIndoor_Crowded	-2.9352081	0.95	-7.851290	2.2728

1-10 of 14 rows | 1-7 of 9 columns Previous **1** 2 Next

5 TBR (Theta/Beta Ratio)

```
hist(scale(eeg_markers_segments_60s$TBR))
```

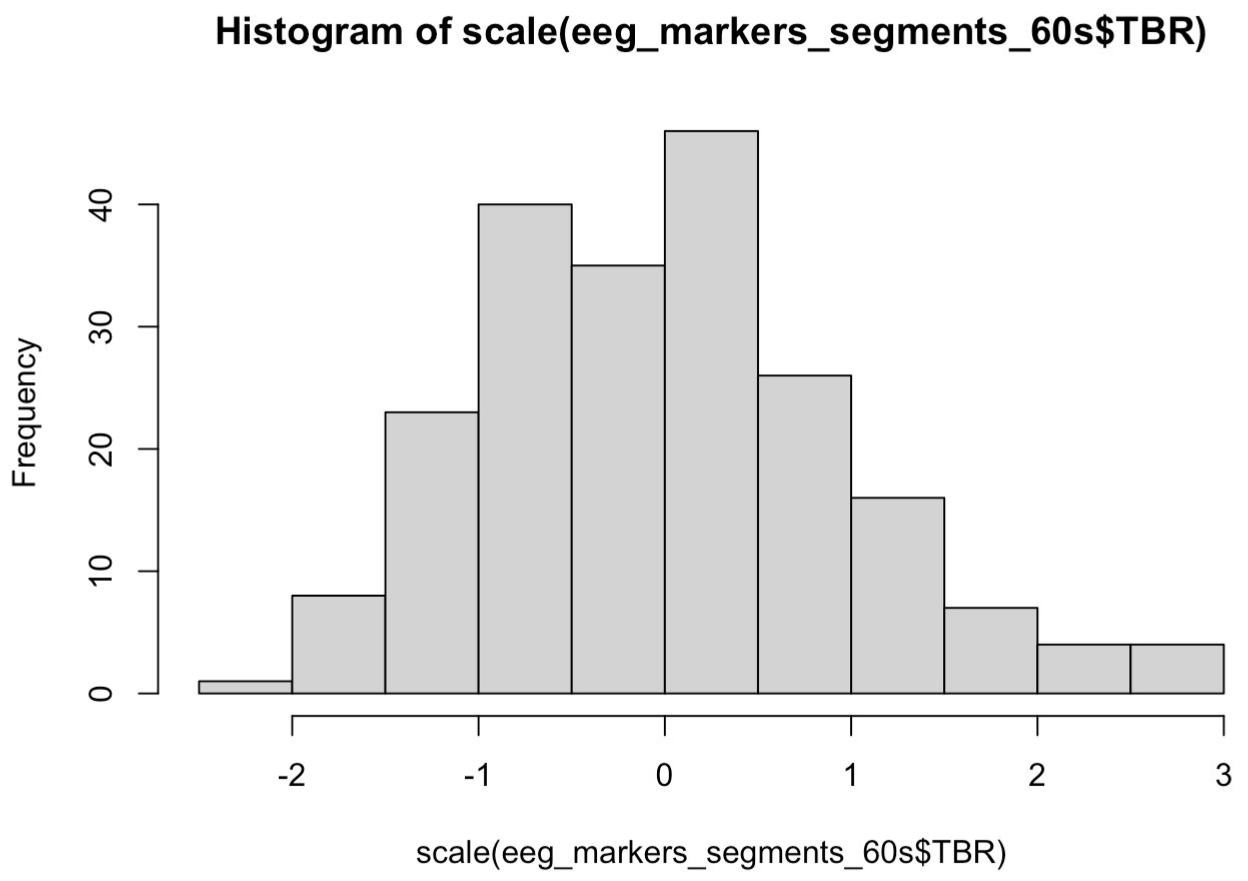


```
bf_tbr_int_student_60s <- brm(formula = bf(TBR ~ Group * Condition + (1 + Condition |
Participant_ID) + (1|schedule), sigma~Group),
                              eeg_markers_segments_60s, #
                          family = student(),
                          iter = 4000,
                          seed = 4343,
                          cores = parallel::detectCores(),
                          prior = set_prior("normal(0,5)"),
                          save_pars = save_pars(all = T),
                          file = here::here("_brmsfiles/bf_tbr_int_student_60s"),
                          control = list(adapt_delta = .999)
)
pp_check(bf_tbr_int_student_60s)
```

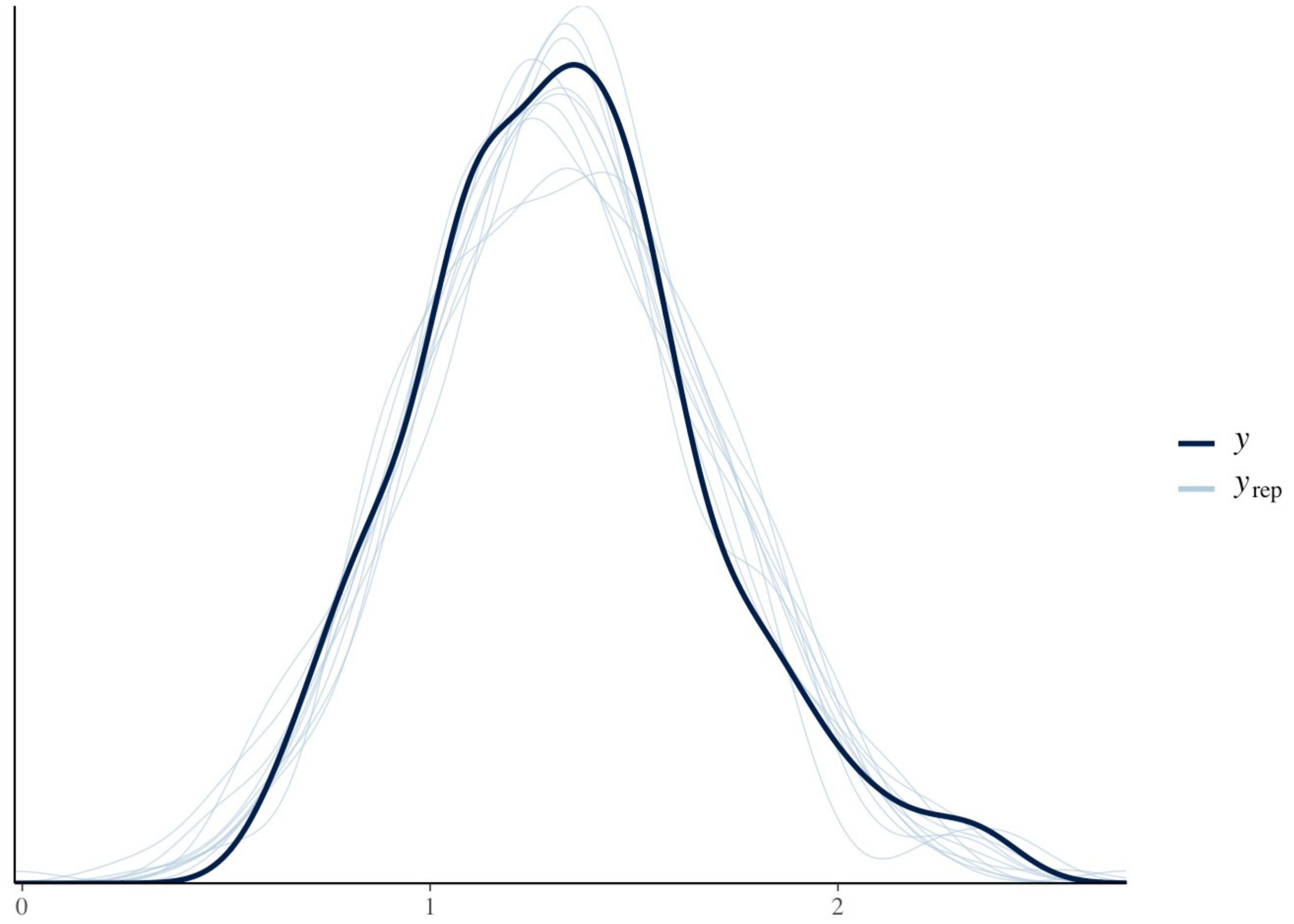

Posterior predictive check (pp_check) looks good.

```
describe_posterior(bf_tbr_int_student_60s, test = "pd")
```

	Parameter <chr>	Median <dbl>	CI <dbl>	CI_low <dbl>	
12	b_Intercept	1.206759948	0.95	-0.04775038	2.7[illegible]
14	b_sigma_Intercept	-1.728935451	0.95	-2.00638906	-1.4[illegible]
6	b_GroupStanding	0.121913514	0.95	-0.10951202	0.3[illegible]
1	b_ConditionGreen_2	0.138815634	0.95	-0.00636794	0.2[illegible]
2	b_ConditionIndoor_Crowded	0.169103658	0.95	0.02691680	0.3[illegible]
3	b_ConditionIndoor_Uncrowded	0.035308337	0.95	-0.09682176	0.1[illegible]
4	b_ConditionOutdoor_Crowded	0.132285251	0.95	-0.03008084	0.2[illegible]
5	b_ConditionOutdoor_Uncrowded	0.061548917	0.95	-0.07527257	0.2[illegible]
7	b_GroupStanding:ConditionGreen_2	-0.044037062	0.95	-0.24664695	0.1[illegible]
8	b_GroupStanding:ConditionIndoor_Crowded	0.065625488	0.95	-0.13175204	0.2[illegible]

1-10 of 16 rows | 1-7 of 9 columns Previous **1** 2 Next

6 FAA(Frontal Alpha Asymmetry)

```
hist(scale(eeg_markers_segments_60s$FAA))
```

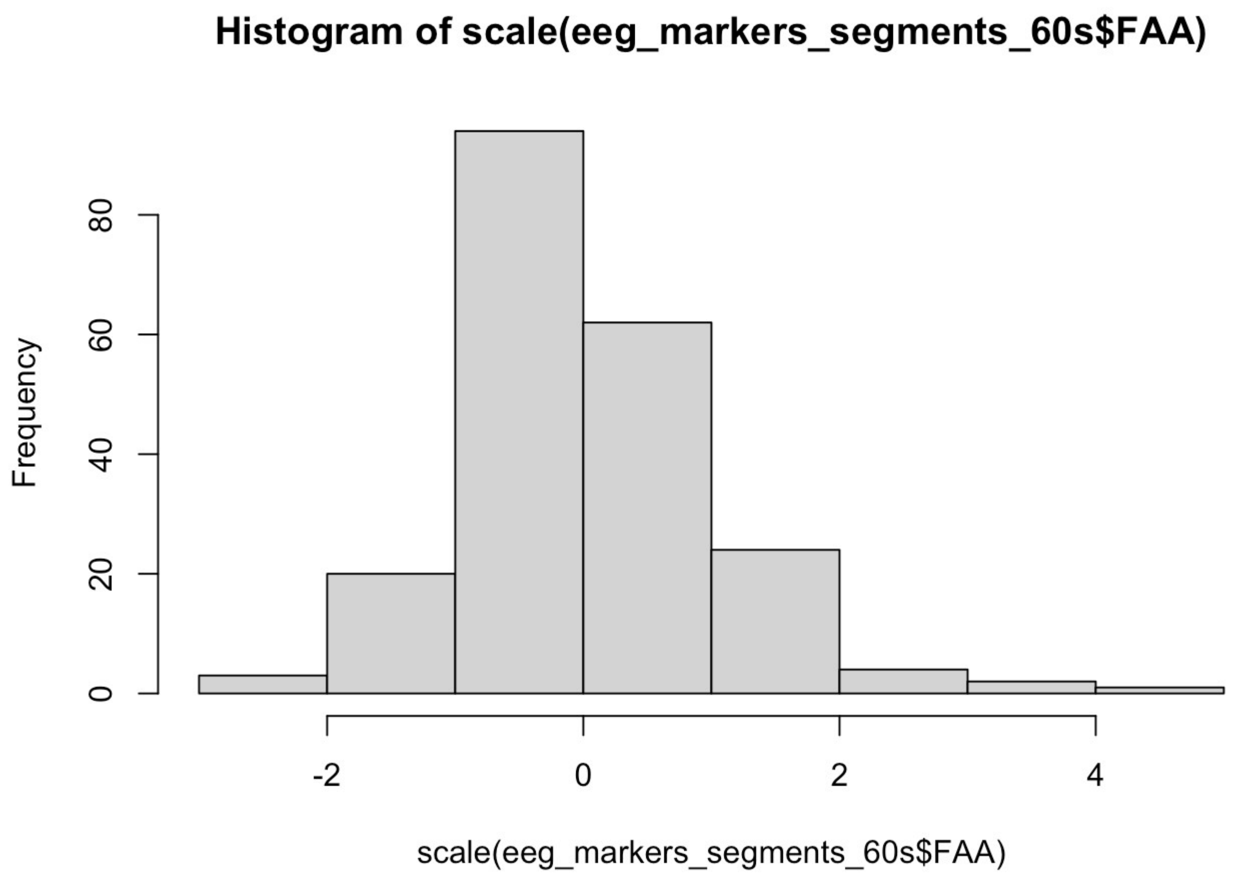


```
bf_faa_int_student_60s <- brm(formula = bf(FAA ~ Group*Condition + (1 + Condition | P
articipant_ID) + (1|schedule), sigma~Group),
                          eeg_markers_segments_60s %>%
                            mutate(FAAs = scale(FAA)) %>%
                            filter(FAAs > -4 & FAAs < 4), # remove one outliers
                          family = student(),
                          iter = 4000,
                          seed = 4343,

                          prior = set_prior("normal(0,5)"),
                          save_pars = save_pars(all = T),
                          file = here::here("_brmsfiles/bf_faa_int_student_60s"),
                          control = list(adapt_delta = .999, max_treedepth  = 15)
)

pp_check(bf_faa_int_student_60s)
```

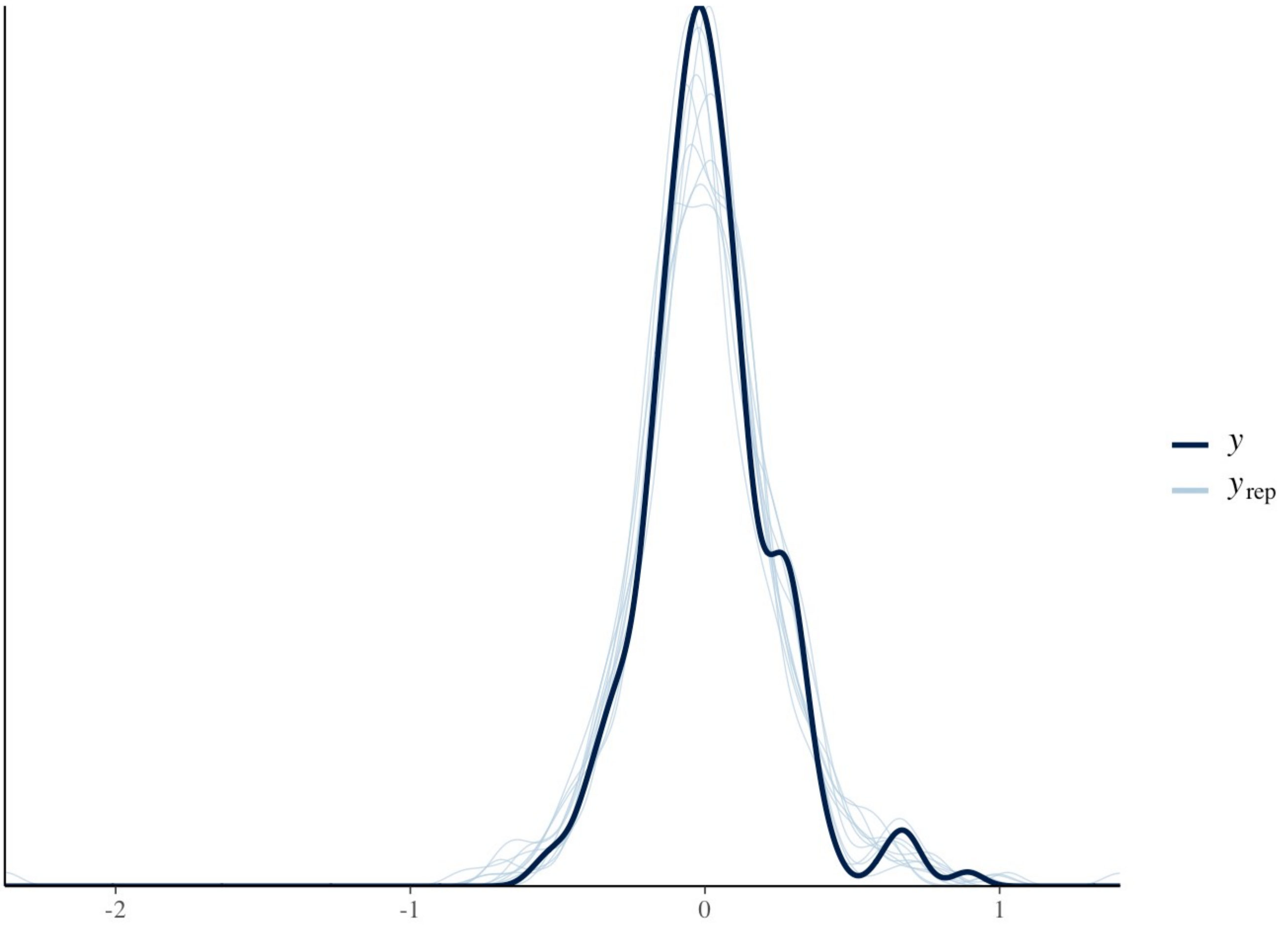


```
loo(bf_faa_int_student_60s)
```

```
##
## Computed from 8000 by 209 log-likelihood matrix
##
##          Estimate   SE
## elpd_loo    125.3 14.7
## p_loo       104.5  6.4
## looic      -250.7 29.3
## ------
## Monte Carlo SE of elpd_loo is NA.
##
## Pareto k diagnostic values:
##                          Count Pct.    Min. n_eff
## (-Inf, 0.5]   (good)     185   88.5%   346
##  (0.5, 0.7]   (ok)        23   11.0%   172
##    (0.7, 1]   (bad)        1    0.5%   101
##    (1, Inf)   (very bad)   0    0.0%   <NA>
## See help('pareto-k-diagnostic') for details.
```

```
describe_posterior(bf_faa_int_student_60s, test = "pd")
```

	Parameter <chr>	Median <dbl>	CI <dbl>	CI_low <dbl>	
12	b_Intercept	-0.009705545	0.95	-1.30285562	1.1
14	b_sigma_Intercept	-2.686018659	0.95	-3.13835381	-2.3
6	b_GroupStanding	-0.033301563	0.95	-0.16802697	0.1
1	b_ConditionGreen_2	0.045792239	0.95	-0.02908506	0.1
2	b_ConditionIndoor_Crowded	0.021916177	0.95	-0.04616117	0.0
3	b_ConditionIndoor_Uncrowded	0.064062422	0.95	-0.00103409	0.1
4	b_ConditionOutdoor_Crowded	0.095930683	0.95	0.03148369	0.1
5	b_ConditionOutdoor_Uncrowded	0.044471550	0.95	-0.01905673	0.1
7	b_GroupStanding:ConditionGreen_2	0.049637412	0.95	-0.07218256	0.1
8	b_GroupStanding:ConditionIndoor_Crowded	-0.026840298	0.95	-0.12742683	0.0

1-10 of 16 rows | 1-7 of 9 columns Previous **1** 2 Next

EDA DATA

Load data

```
eda_videobaseline_delta_60s <- readRDS("~/Nextcloud/VR_LAB_EDA_VAL/EDA_VALIDATION/ana
lysis/_data/eda_video-baseline_delta_60s.rds")
eda_videobaseline_delta_60s
```

Participant_ID <chr>	Group <chr>	Condition <chr>	d_Tonic_mean <dbl>	d_ISCR <dbl>	d_n... <dbl>	d_Am
1302	Standing	Green_1	-1.102674619	4.84295623	0	1.177
1302	Standing	Indoor_Crowded	-1.150777704	-18.24476146	-8	-4.985
1302	Standing	Outdoor_Uncrowded	-0.659167603	0.43181911	-3	-0.125
1302	Standing	Green_2	-0.851794694	1.98848182	0	-0.161
1302	Standing	Outdoor_Crowded	-0.991517353	-4.44801743	-3	-0.724
1302	Standing	Indoor_Uncrowded	-1.079973445	-8.46157806	-7	-1.980
1370	Standing	Indoor_Uncrowded	-1.422206718	-0.91470041	2	-0.056
1370	Standing	Outdoor_Crowded	-0.027549123	16.54284728	19	4.277
1370	Standing	Green_1	-2.291949412	-7.96299654	-4	-3.065
1370	Standing	Outdoor_Uncrowded	-1.680977767	1.63054070	1	0.533

1-10 of 192 rows | 1-7 of 8 columns Previous 1 2 3 4 5 6 ... 20 Next

7 Tonic EDA

```
eda_videobaseline_delta_60s %>%
  ggplot(aes(d_Tonic_mean, colour = Group)) +
  geom_density(outline.type = "upper") +
  facet_wrap(.~Condition)
```

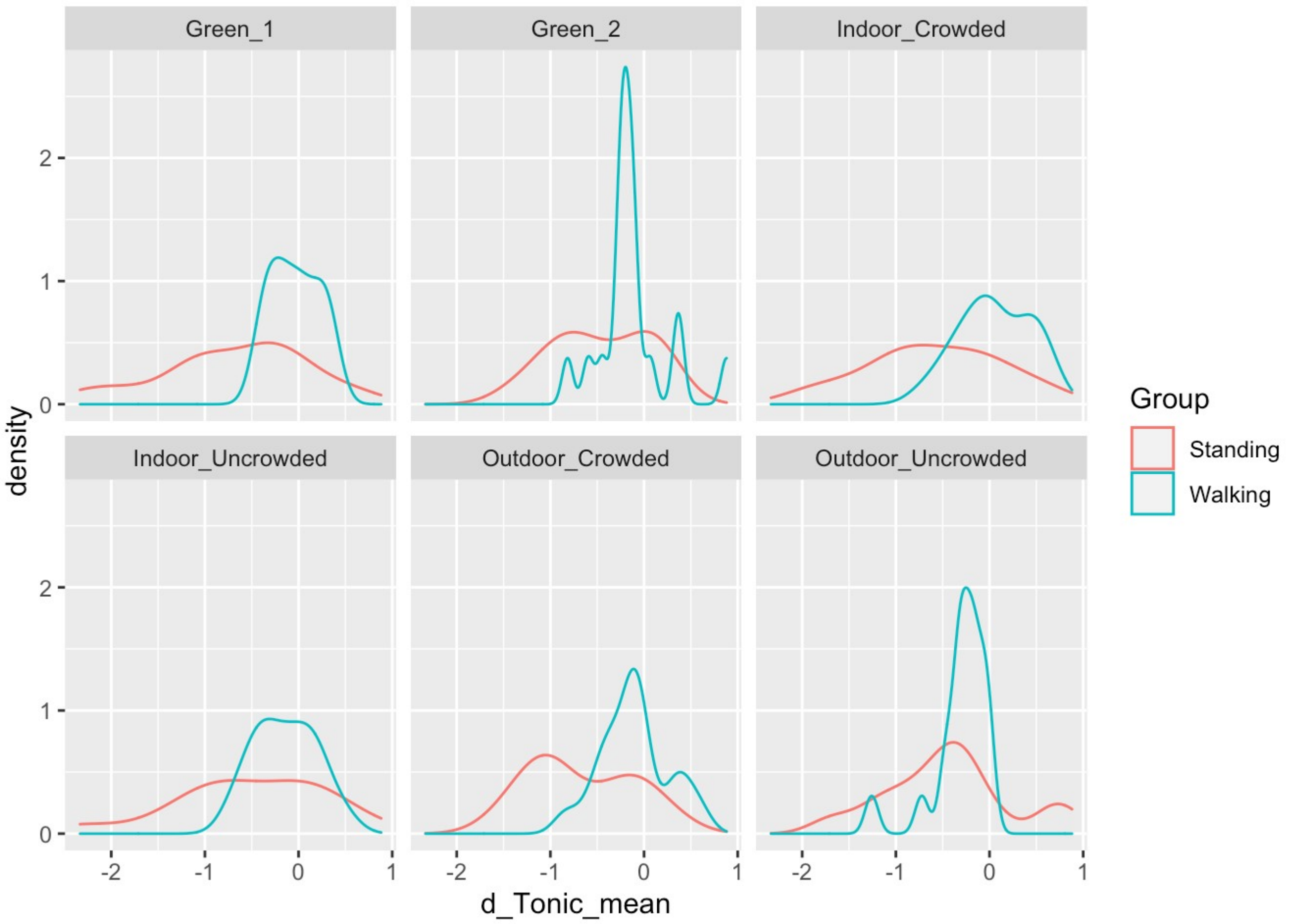

```
bf_tonic_int_student <- brm(bf(d_Tonic_mean ~ Group * Condition + (1 + Condition | Pa
rticipant_ID) + (1 | schedule), sigma ~ Group),
                                    data = eda_videobaseline_delta_60s,
                                    family = student(),
                                    iter = 4000,
                                    seed = 4343,
                                    prior = set_prior("normal(0,10)"),
                                    save_pars = save_pars(all = T),
                                    file = here::here("_brmsfiles/bf_tonic_int_studen
t"),
                                    control = list(adapt_delta = .99, max_treedepth  =
15)
                                    )
pp_check(bf_tonic_int_student)
```

```
## Using 10 posterior draws for ppc type 'dens_overlay' by default.
```

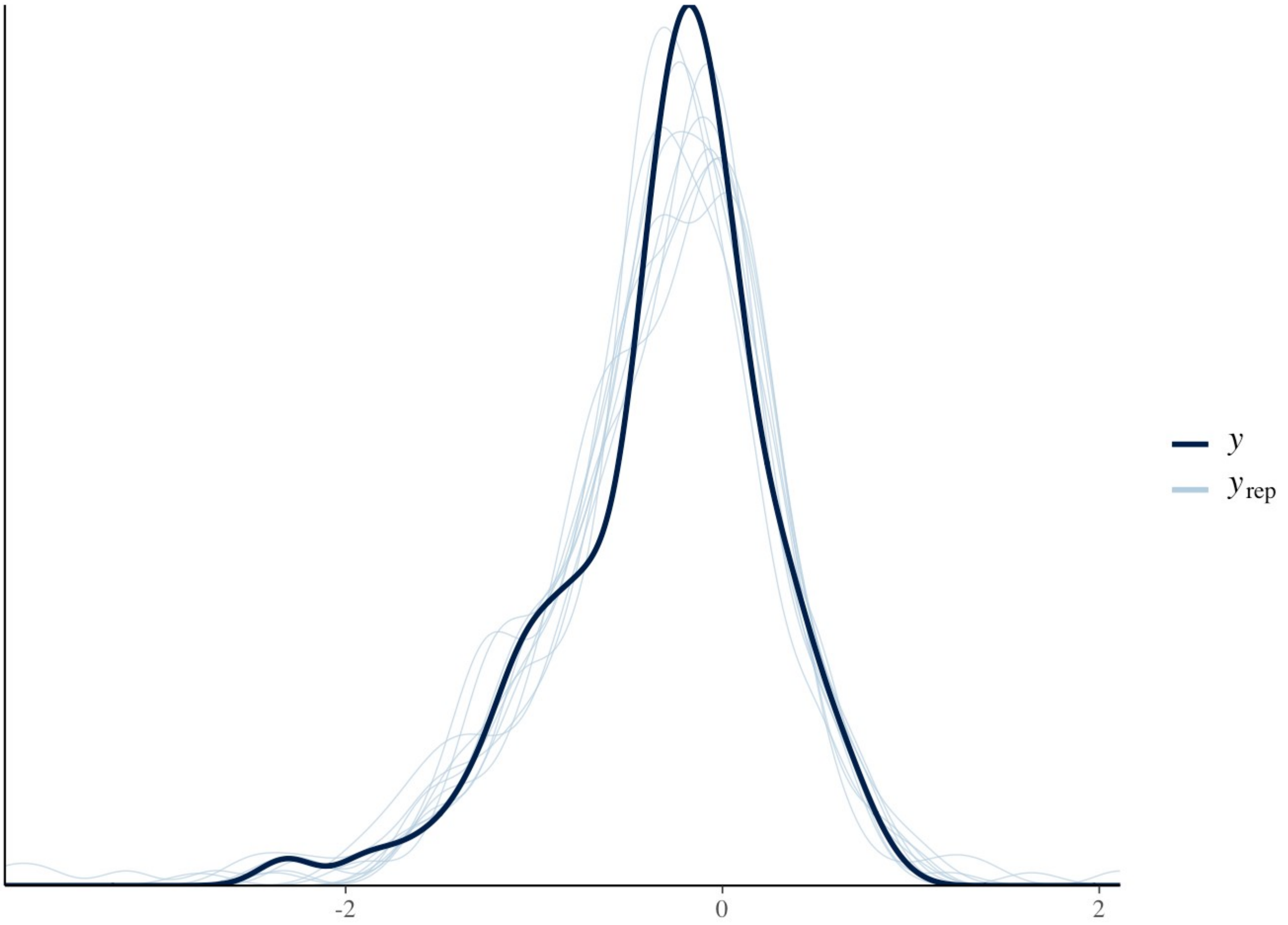


```
describe_posterior(bf_tonic_int_student, test = "pd")
```

Parameter	Median	CI	CI_low
<chr>	<dbl>	<dbl>	<dbl>

	Parameter <chr>	Median <dbl>	CI <dbl>	CI_low <dbl>	
12	b_Intercept	-0.6691144028	0.95	-2.1801430	1.06
14	b_sigma_Intercept	-0.5922147406	0.95	-0.9021014	-0.33
6	b_GroupWalking	0.6730286588	0.95	0.2934697	1.02
1	b_ConditionGreen_2	0.2184769755	0.95	-0.2353993	0.67
2	b_ConditionIndoor_Crowded	0.0659901474	0.95	-0.3795858	0.54
3	b_ConditionIndoor_Uncrowded	0.1795261511	0.95	-0.3038618	0.61
4	b_ConditionOutdoor_Crowded	0.0009971214	0.95	-0.4315166	0.46
5	b_ConditionOutdoor_Uncrowded	0.1880749350	0.95	-0.2322233	0.64
7	b_GroupWalking:ConditionGreen_2	-0.3240237349	0.95	-0.8300768	0.16
8	b_GroupWalking:ConditionIndoor_Crowded	0.0354771751	0.95	-0.4453340	0.55

1-10 of 16 rows | 1-7 of 9 columns Previous **1** 2 Next

8 ISCR EDA

```
eda_videobaseline_delta_60s %>%
  mutate(d_ISCR_s = scale(d_ISCR)) %>%
  filter(d_ISCR_s > -4 & d_ISCR_s < 4) %>%
  ggplot(aes(d_ISCR, colour = Group)) +
  geom_density(outline.type = "upper") +
  facet_wrap(.~Condition)
```

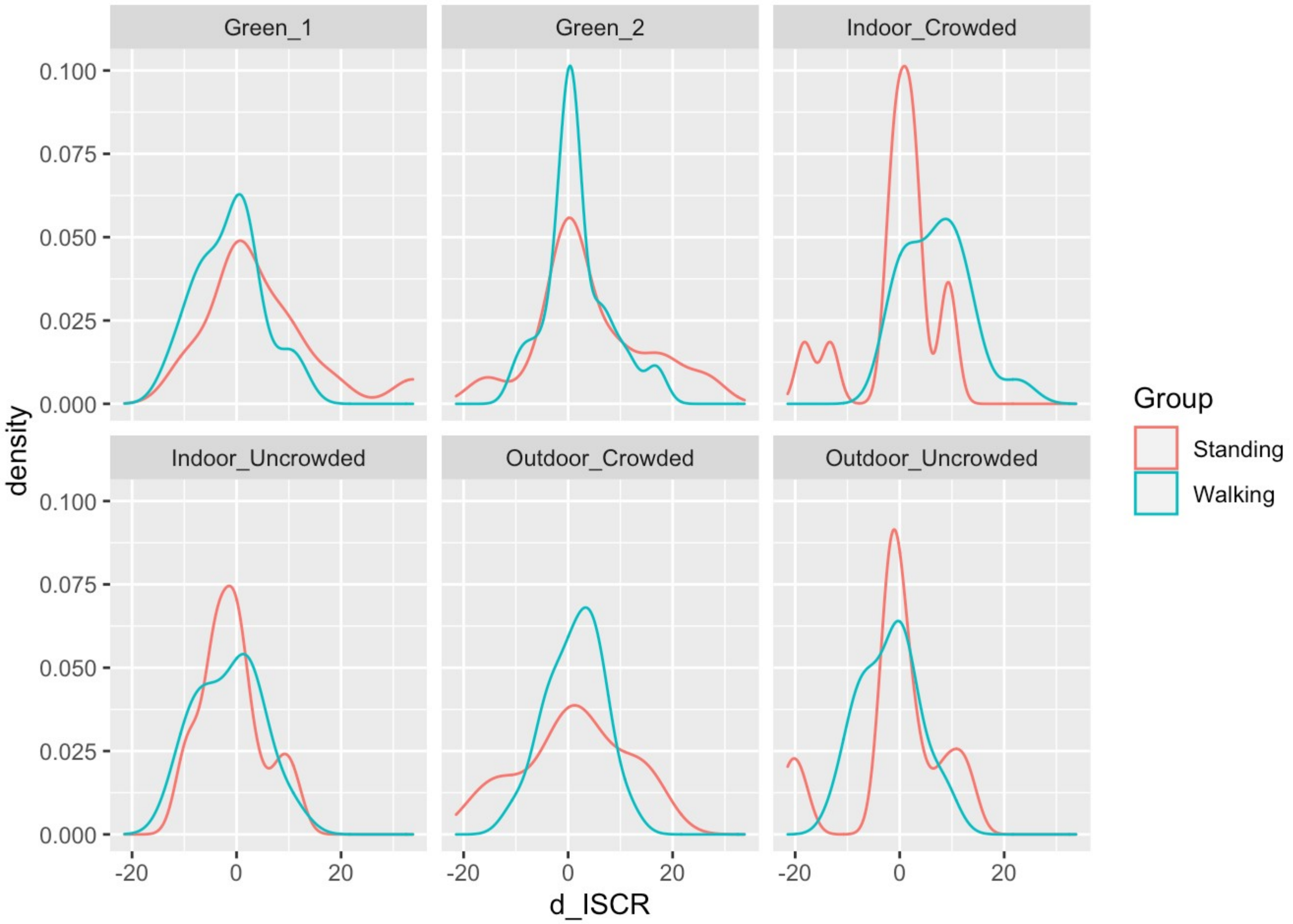


```
bf_iscr_int_student <- brm(bf(d_ISCR ~ Group * Condition + (1 + Condition|Participant
_ID) + (1|schedule), sigma ~ Group),
                                  data = eda_videobaseline_delta_60s,
                                  family = student(),
                                  iter = 4000,
                                  seed = 4343,
                                  prior = set_prior("normal(0,10)"),
                                  save_pars = save_pars(all = T),
                                  file = here::here("_brmsfiles/bf_iscr_int_student"),
                                  control = list(adapt_delta = .999)
                            )

pp_check(bf_iscr_int_student)
```

```
## Using 10 posterior draws for ppc type 'dens_overlay' by default.
```

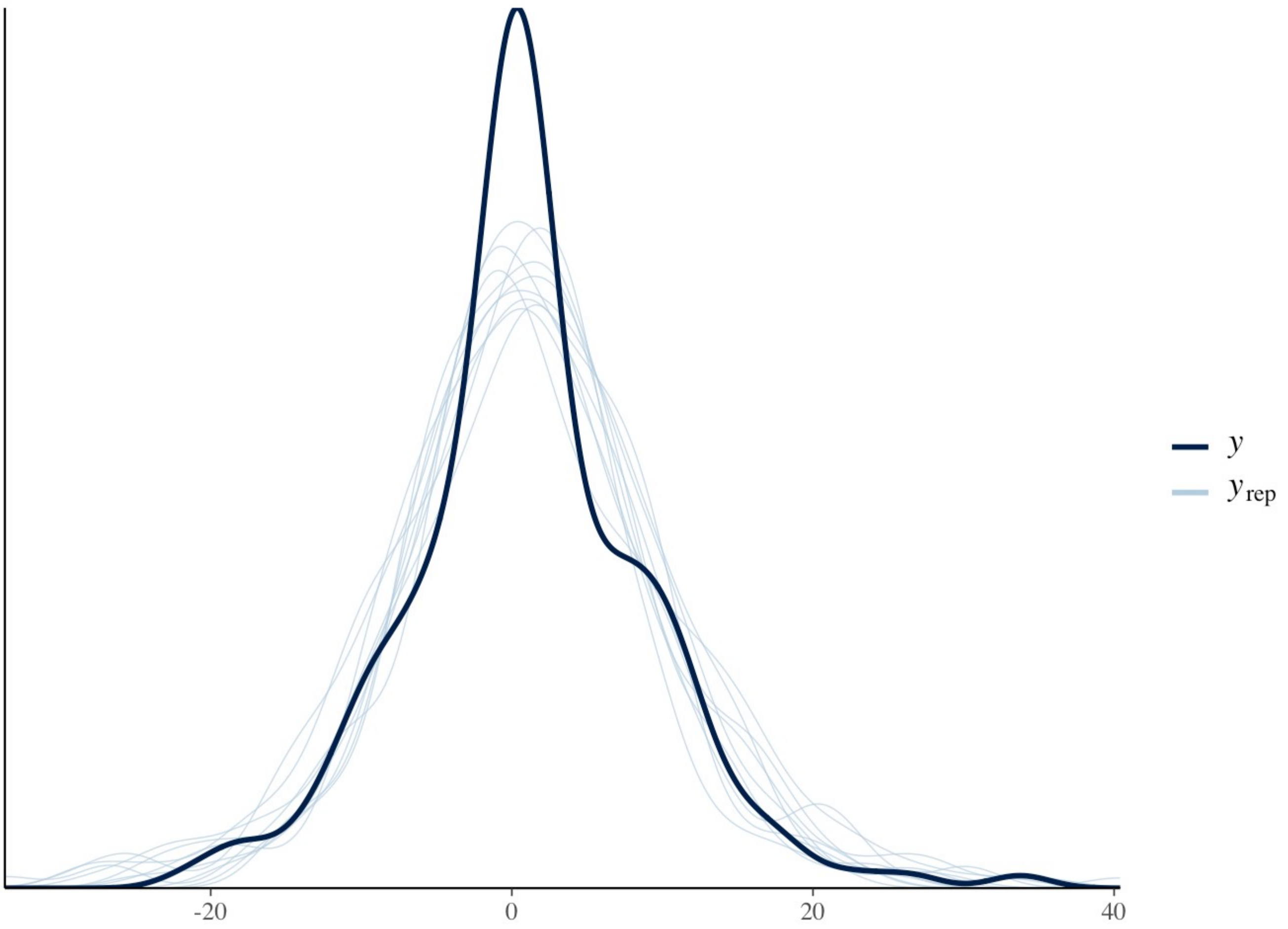


```
describe_posterior(bf_iscr_int_student, test = "pd")
```

	Parameter <chr>	Median <dbl>	CI <dbl>	CI_low <dbl>	CI_high <dbl>
12	b_Intercept	2.2452477	0.95	-3.684022	8.2536973
14	b_sigma_Intercept	2.1314965	0.95	1.912115	2.3282676
6	b_GroupWalking	-2.9013975	0.95	-7.403174	1.8996067
1	b_ConditionGreen_2	1.6433287	0.95	-4.196037	7.4795432
2	b_ConditionIndoor_Crowded	-1.7875911	0.95	-7.446361	4.1094716
3	b_ConditionIndoor_Uncrowded	-3.0325713	0.95	-8.532996	2.8310099
4	b_ConditionOutdoor_Crowded	-0.9687282	0.95	-6.652783	4.9031908
5	b_ConditionOutdoor_Uncrowded	-2.6303674	0.95	-8.282850	3.1063347
7	b_GroupWalking:ConditionGreen_2	0.9172300	0.95	-6.111678	7.0552508
8	b_GroupWalking:ConditionIndoor_Crowded	9.5235418	0.95	2.805445	15.9890010

1-10 of 16 rows | 1-7 of 9 columns Previous **1** 2 Next

Session information

R and packages used

```
sessionInfo()
```

```
## R version 4.1.1 (2021-08-10)
## Platform: x86_64-apple-darwin17.0 (64-bit)
## Running under: macOS Catalina 10.15.7
##
## Matrix products: default
## BLAS:   /Library/Frameworks/R.framework/Versions/4.1/Resources/lib/libRblas.0.dyli
b
## LAPACK: /Library/Frameworks/R.framework/Versions/4.1/Resources/lib/libRlapack.dyli
b
##
## locale:
## [1] en_US.UTF-8/en_US.UTF-8/en_US.UTF-8/C/en_US.UTF-8/en_US.UTF-8
##
## attached base packages:
## [1] stats     graphics  grDevices utils     datasets  methods   base
##
## other attached packages:
##  [1] bayestestR_0.11.0 patchwork_1.1.1   brms_2.16.2       Rcpp_1.0.7
##  [5] forcats_0.5.1     stringr_1.4.0     dplyr_1.0.7       purrr_0.3.4
##  [9] readr_2.0.1       tidyr_1.1.3       tibble_3.1.5      ggplot2_3.3.5
## [13] tidyverse_1.3.1
##
## loaded via a namespace (and not attached):
##   [1] readxl_1.3.1         backports_1.2.1      plyr_1.8.6
##   [4] igraph_1.2.6         splines_4.1.1        crosstalk_1.1.1
##   [7] rstantools_2.1.1     inline_0.3.19        digest_0.6.28
##  [10] htmltools_0.5.2      rsconnect_0.8.24     fansi_0.5.0
##  [13] magrittr_2.0.1       checkmate_2.0.0      tzdb_0.1.2
##  [16] modelr_0.1.8         RcppParallel_5.1.4   matrixStats_0.61.0
##  [19] vroom_1.5.4          xts_0.12.1           prettyunits_1.1.1
##  [22] colorspace_2.0-2     rvest_1.0.1          haven_2.4.3
##  [25] xfun_0.26            callr_3.7.0          crayon_1.4.1
##  [28] jsonlite_1.7.2       lme4_1.1-27.1        survival_3.2-11
##  [31] zoo_1.8-9            glue_1.4.2           gtable_0.3.0
##  [34] emmeans_1.7.0        V8_3.4.2             distributional_0.2.2
##  [37] pkgbuild_1.2.0       rstan_2.26.3         abind_1.4-5
##  [40] scales_1.1.1         mvtnorm_1.1-3        DBI_1.1.1
##  [43] miniUI_0.1.1.1       xtable_1.8-4         bit_4.0.4
##  [46] stats4_4.1.1         StanHeaders_2.26.3   DT_0.19
##  [49] datawizard_0.2.0     htmlwidgets_1.5.4    httr_1.4.2
##  [52] threejs_0.3.3        posterior_1.1.0      ellipsis_0.3.2
##  [55] pkgconfig_2.0.3      loo_2.4.1            farver_2.1.0
##  [58] sass_0.4.0           dbplyr_2.1.1         here_1.0.1
##  [61] utf8_1.2.2           labeling_0.4.2       tidyselect_1.1.1
##  [64] rlang_0.4.11         reshape2_1.4.4       later_1.3.0
##  [67] munsell_0.5.0        cellranger_1.1.0     tools_4.1.1
##  [70] cli_3.0.1            generics_0.1.0       broom_0.7.9
##  [73] ggridges_0.5.3       evaluate_0.14        fastmap_1.1.0
##  [76] yaml_2.2.1           bit64_4.0.5          processx_3.5.2
##  [79] knitr_1.33           fs_1.5.0             nlme_3.1-152
##  [82] mime_0.12            projpred_2.0.2       rstanarm_2.21.1
```

```
##  [85] xml2_1.3.2          compiler_4.1.1       bayesplot_1.8.1
##  [88] shinythemes_1.2.0   rstudioapi_0.13      curl_4.3.2
##  [91] gamm4_0.2-6         reprex_2.0.1         bslib_0.3.1
##  [94] stringi_1.7.5       highr_0.9            ps_1.6.0
##  [97] Brobdingnag_1.2-6   lattice_0.20-44      Matrix_1.3-4
## [100] nloptr_1.2.2.2      markdown_1.1         shinyjs_2.0.0
## [103] tensorA_0.36.2      vctrs_0.3.8          pillar_1.6.3
## [106] lifecycle_1.0.1     jquerylib_0.1.4      bridgesampling_1.1-2
## [109] estimability_1.3    insight_0.14.3       httpuv_1.6.3
## [112] R6_2.5.1            promises_1.2.0.1     gridExtra_2.3
## [115] codetools_0.2-18    boot_1.3-28          colourpicker_1.1.1
## [118] MASS_7.3-54         gtools_3.9.2         assertthat_0.2.1
## [121] rprojroot_2.0.2     withr_2.4.2          shinystan_2.5.0
## [124] mgcv_1.8-36         parallel_4.1.1       hms_1.1.0
## [127] grid_4.1.1          coda_0.19-4          minqa_1.2.4
## [130] rmarkdown_2.10      shiny_1.7.1          lubridate_1.7.10
## [133] base64enc_0.1-3     dygraphs_1.1.1.6
```
